# Genes Selectively Up-Regulated by Pheromone in White Cells Are Involved in Biofilm Formation in *Candida albicans*

**DOI:** 10.1371/journal.ppat.1000601

**Published:** 2009-10-02

**Authors:** Nidhi Sahni, Song Yi, Karla J. Daniels, Thyagarajan Srikantha, Claude Pujol, David R. Soll

**Affiliations:** Department of Biology, The University of Iowa, Iowa City, Iowa, United States of America; University of Wisconsin, Madison, United States of America

## Abstract

To mate, *MTL*-homozygous strains of the yeast pathogen *Candida albicans* must switch from the white to opaque phase. Mating-competent opaque cells then release pheromone that induces polarization, a G1 block and conjugation tube formation in opaque cells of opposite mating type. Pheromone also induces mating-incompetent white cells to become adhesive and cohesive, and form thicker biofilms that facilitate mating. The pheromone response pathway of white cells shares the upstream components of that of opaque cells, but targets a different transcription factor. Here we demonstrate that the genes up-regulated by the pheromone in white cells are activated through a common *cis*-acting sequence, WPRE, which is distinct from the *cis*-acting sequence, OPRE, responsible for up-regulation in opaque cells. Furthermore, we find that these white-specific genes play roles in white cell biofilm formation, and are essential for biofilm formation in the absence of an added source of pheromone, suggesting either an autocrine or pheromone-independent mechanism. These results suggest an intimate, complex and unique relationship between switching, mating and *MTL*-homozygous white cell biofilm formation, the latter a presumed virulence factor in *C. albicans*.

## Author Summary

*Candida albicans*, like other microbial pathogens, form protective biofilms on host tissue, prosthetics and catheters. But *C. albicans* forms two types of biofilm, one by cells of majority strains that are heterozygous at the mating type locus, and another by white cells of minority strains that are homozygous at the mating type locus. These latter biofilms are enhanced by mating-competent minority opaque cells, a source of pheromone. The white cell biofilm response to pheromone is regulated by a pheromone response pathway that shares all of the upper components of the opaque cell mating response pathway, but targets a different transcription factor and activates different phase-specific downstream genes. Here we demonstrate that genes are up-regulated by pheromone in white cells through a common white *cis*-acting specific pheromone response element (WPRE), distinct from the element (OPRE) responsible for pheromone up-regulation of genes in opaque cells. In addition, the pheromone-induced white-specific genes play essential roles in biofilm formation. We further demonstrate that in the absence of minority opaque cells, the source of pheromone, majority white cells form biofilms through a process that is still dependent upon the same pheromone response pathway and targeted white-specific genes, suggesting either an autocrine system that involves the auto release of pheromone of the opposite mating type, or pheromone-independent activation. These observations indicate a unique interdependency of white cell biofilm formation, a presumed pathogenic trait, switching and mating in *C. albicans*.

## Introduction

In nature, the majority of natural strains of *Candida albicans* are heterozygous (**a**/α) at the mating type locus, *MTL* [1]–[4]. To mate, they must first undergo homozygosis to **a**/**a** or α/α [5]–[7], then switch from the white to unique opaque phenotype [8],[9]. Switching from the white to opaque phenotype occurs spontaneously and involves activation and deactivation of the master switch locus, *WOR1* [10]–[12].

In the mating process, opaque cells of each mating type release their respective pheromone, which induces opaque cells of the opposite mating type to polarize, form a mating projection, become blocked in G1, undergo chemotropism and fuse, in a fashion similar to that of haploid cells of *Saccharomyces cerevisiae* [9], [13]–[19]. The requirement to switch from white to opaque, however, was considered both unique and paradoxical. Why did *C. albicans* undergo a phenotypic transition that was so highly complex in order to become mating-competent, when there was no similar requirement for *S. cerevisiae*? The surprising discovery that mating-incompetent white cells also responded to the mating pheromone, but in a manner different from that of mating-competent opaque cells, provided a clue. Pheromone induced none of the responses in white cells that it induced in opaque cells. Rather, it induced cohesion between cells, adhesion to a substratum and enhanced biofilm development [20]. Biofilm formation is a general virulence factor of both prokaryotic and eukaryotic microbes that provides environments resistant to antibodies and host challenges [21]–[23]. In the case of biofilms formed by *C. albicans* white cells, it also provides an environment that facilitates mating [20]. The white cell response to pheromone has been shown to be a general characteristic of *MTL*-homozygous strains for all of the major clades of *C. albicans*, including derivatives of the laboratory strain SC5314 [24].

Mutational analyses have revealed that the white cell response to pheromone involves the same receptors and MAP-kinase pathway as the opaque cell mating response [25],[26]. This pathway, however, activates a different downstream *trans*-acting factor [25]. Deletion of *CPH1*, which encodes the downstream transcription factor targeted by the pheromone response pathway in opaque cells, blocks the opaque cell response, but not the white cell response [25]. The downstream transcription factor targeted by the pheromone response pathway in white cells has not yet been identified. In white cells pheromone induces the expression of a number of genes that are also induced in opaque cells, as well as a number of genes specific to white cells [13],[20],[24]. Since the major effects of pheromone on white cells includes increased adhesion, increased cohesion and enhanced biofilm formation, we predicted that pheromone-induced white-specific genes would play key roles in these processes. Here we demonstrate that white-specific genes are regulated through an A-rich white-specific pheromone response element, WPRE (AAAAAAAAAAGAAAG), which is distinct from the G-rich response element, OPRE (GTGAGGGGA), regulating genes in the opaque cell pheromone response. These results support our earlier conclusion that white genes are regulated by a single white-specific *trans*-acting factor [25]. Furthermore, we show by mutational analysis that the white-specific genes play fundamental roles in adhesion and white cell biofilm formation. Moreover, these genes as well as the signal transduction pathway, are essential for white cell biofilm formation in the absence of an opaque cell pheromone source, indicating either the existence of a pheromone-based autocrine system, or a pheromone-independent process. Interestingly, the white-specific genes up-regulated by pheromone in *MTL*-homozygous white cells have previously been demonstrated to play roles in biofilm formation in **a**/α cells, which represent a majority of the strains found in nature [1]–[4]. Together, these results provide clues to the evolution of the white cell pheromone response.

## Results

### Selective induction of white-specific genes

**Figure 1:**
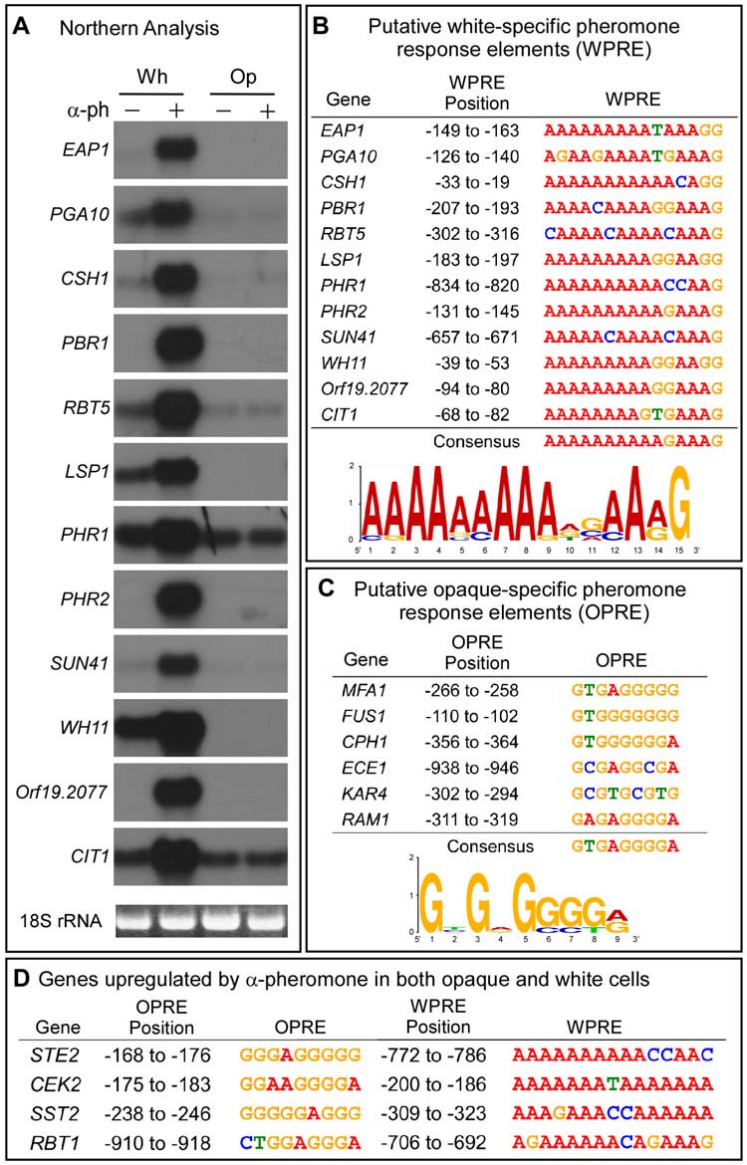
Twelve genes were identified that were strongly up-regulated by α-pheromone in white a/a, but not opaque a/a, cells. Each of these genes contained one or more putative white-specific pheromone response elements (WPRE) in their promoters. A. Northern analysis of the expression of twelve genes in white and opaque a/a cells in the absence (−) and presence (+) of α-pheromone (α-ph) that were identified in a screen of 103 genes as strongly up-regulated. B. The sequence considered to represent the putative white-specific pheromone response element (WPRE) in the promoters of the 12 selected white-specific genes in panel A, using a high stringency E value threshold of ≤0.001 in the Multiple for Motif Elicitation (MEME) software. The consensus sequence for WPRE is given at the bottom of the panel. C. The sequence considered the putative opaque-specific pheromone response element (OPRE) in the promoters of six genes selectively up-regulated by pheromone in opaque cells, using a threshold of 0.001 in the MEME program. The consensus sequence for OPRE is presented at the bottom of the panel. D. Genes up-regulated by α-pheromone in both opaque and white cells contain both OPRE and WPRE. The positions of the OPRE and WPRE sequence with the highest homology to the consensus sequence is given relative to the start codon in panels B, C and D.

In past studies, the genes *CSH1* [25], orf19.2077 [24] and orf19.6274 [24] had been demonstrated to be selectively up-regulated by pheromone in white, but not opaque, cells. To identify additional genes similarly up-regulated, we analyzed by northern blot hybridization the expression patterns of 103 genes that had been implicated in adhesion, cell wall biogenesis, biofilm formation, filamentation or switching (supplemental Table S1). Nine of these genes (*EAP1*, *PGA10*, *RBT5*, *PHR1*, *PHR2*, *LSP1*, *CIT1*, *SUN41*, *WH11*) were strongly up-regulated in white but not opaque cells (Figure 1A). With the genes *CSH1*, orf19.2077 and orf19.6274 (Figure 1A), the last renamed *PBR1* (Pheromone-induced Biofilm Regulator 1) for its role in white cell biofilm formation, which we demonstrate here, we had 12 genes for further analysis that were selectively up-regulated by pheromone in white but not opaque cells.

### Putative pheromone-regulated *cis*-acting elements

To identify potential pheromone-regulated *cis*-acting elements, the promoters of the 12 white-specific test genes and the promoters of six genes that had previously been shown to be selectively up-regulated by pheromone in opaque cells, *MFA1*, *FUS1*, *CPH1*, *ECE1*, *KAR4* and *RAM1* ([14],[20],[25],[27] and N. Sahni, S. Yi, D. R. Soll, unpublished observations), were subjected to sequence analysis with the Multiple EM (model) for Motif Elicitation (MEME) software [28]–[30] in order to identify among the white group and among the opaque group consensus sequences with the highest level of homology. The one thousand base pair upstream regions of the genes in the white and opaque sets were each analyzed for a common motif with an upper length limit of 15 bp at an E value of ≤0.001 as a threshold [28]–[30]. At this stringent threshold, the E value represents the expected number of motifs with a score as good or better than the analyzed motif, in a set of similar sized random sequences [30]. The promoters of all 12 white-specific genes contained at least one copy of a putative white pheromone-regulated element (WPRE) with high homology to the consensus sequence AAAAAAAAAAGAAAG (Figure 1B; supplemental Table S5). Using the same stringent E value of ≤0.001 as a threshold, this DNA sequence was found to be absent in the promoters of the six genes selectively up-regulated by pheromone in opaque but not white cells. The consensus sequence had no homology to the consensus sequences for the pheromone response elements (PRE) of *S. cerevisiae* [31]–[33] or *Ustilago maydis* [34]. The white-specific gene promoters also contained WPRE-like sequences with lower homology to the consensus sequence (supplemental Table S5). Only the WPRE sequence with the highest homology to the consensus sequence was included for initial analyses.

Again using the MEME program at an E value of ≤0.001 as a threshold and with an upper length limit of 15 bp [28]–[30], the promoters of all six opaque-specific genes were found to contain at least one copy of a putative opaque pheromone-regulated element (OPRE) with the unique consensus sequence GTGAGGGGA (Figure 1C; supplemental Table S6). At this E value, this element was absent in the promoters of the 12 genes selectively up-regulated by pheromone in white cells. It exhibited no significant homology with the PREs of mating genes in *S. cerevisiae* [31]–[33] or *U. maydis* [34]. Bennett and Johnson [27] had reported the presence of a putative PRE element similar to that in *S. cerevisiae* in some, but not all, pheromone up-regulated genes in opaque cells. In an expanded list of ten pheromone-regulated opaque genes (*MFA1*, *FUS1*, *STE2*, *SST2*, *CPH1*, *KAR4*, *ECE1*, *RAM1*, *CEK2*, *RBT1*), we found sequences weakly homologous to the *S. cerevisiae* PRE-like element with an average E value of 10^+2^, which indicates that the homology is probably spurious [28]–[30]. In contrast, every one of the 10 genes had an OPRE sequence with an average E value of 10^−5^ (supplemental Table S6). Decreasing the length in the MEME search to a maximum of nine base pairs identified the same consensus sequences (supplemental Table S6) These results strongly suggested that the OPREs were better candidates for a *cis*-acting sequence regulating pheromone-induced expression of opaque genes than sequences weakly homologous to the *S. cerevisiae* PRE-like sequences, which were weakly to negligibly homologous to each other and not present in all opaque genes regulated by pheromone (supplemental Table S6).

If pheromone up-regulated opaque-specific genes through OPRE and white-specific genes through WPRE, then genes up-regulated by pheromone in both opaque and white cells should have both elements with the same high E values of white- and opaque-specific genes. Four such genes, *STE2*, *CEK2*, *SST2* and *RBT1* [20],[27], were analyzed by MEME software using the same high stringency E value of ≤0.001 as a threshold. The promoters of all four genes had at least one WPRE and at least one OPRE at this stringent threshold (Figure 1D; supplemental Table S5 and S6).

### WPRE regulates pheromone-induced white-specific gene expression

Although sequence analyses revealed potential *cis*-acting elements, only functional analyses can establish their roles as such. To test whether the putative WPRE functioned as a pheromone-responsive *cis*-acting sequence for white-specific genes, one allele of each of the four white-specific genes *EAP1*, *PGA10*, *CSH1* and *PBR1* selected from the group of 12 genes, was deleted in the natural **a**/**a** strain P37005 to generate the heterozygous deletion mutants *EAP1*/*eap1*, *PGA10*/*pga10*, *CSH1*/*csh1* and *PBR1*/*pbr1*. The WPRE in the promoter of the retained allele in each heterozygote with the highest homology to the WPRE consensus sequence was then selectively deleted, resulting in the WPRE deletion mutants *EAP1_WPREΔ_*/*eap1*, *PGA10_WPREΔ_*/*pga10*, *CSH1_WPREΔ_*/*csh1* and *PBR1_WPREΔ_*/*pbr1*. The WPRE deletion derivative of each of these mutants was then replaced with the native gene and promoter to generate the complemented controls *EAP1_WPREΔ_-EAP1*/*eap1*, *PGA10_WPREΔ_-PGA10*/*pga10*, *CSH1_WPREΔ_-CSH1*/*csh1* and *PBR1_WPREΔ_-PBR1*/*pbr1*. All complemented strains contained a GFP tag at the 3′ end of the open reading frame for protein localization studies and western analysis. The homozygous deletion mutants *eap1/eap1*, *pga10/pga10*, *csh1/csh1* and *pbr1/pbr1*, were also created by deleting the remaining alleles in the original heterozygous mutant.

**Figure 2:**
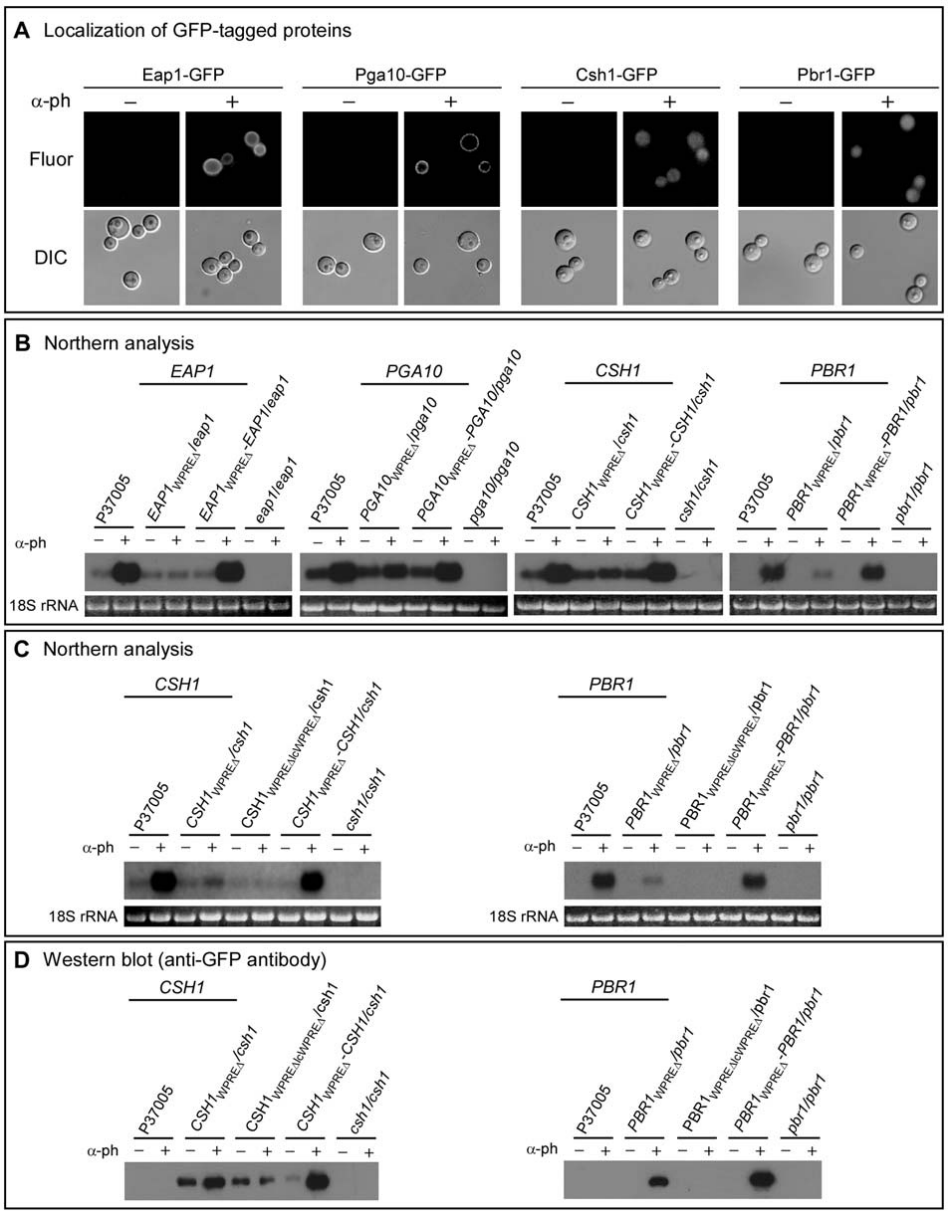
Localization of Eap1, Pga10, Csh1 and Pbr1 and the role of WPRE in the induction of transcription. A. GFP visualization reveals that Eap1 and Pga10 localize primarily to the cell surface, and Csh1 and Pbr1 localize primarily in the cytosol upon induction by α-pheromone. The complemented strains *EAP1*_WPREΔ_-*EAP1/eap1*, *PGA10*_WPREΔ_-*PGA10/pga10*, *CSH1*_WPREΔ_-*CSH1/csh1* and *PBR1*_WPREΔ_-*PBR1/pbr1*, which were tagged at the carboxy terminus with GFP, were examined. B. Northern analysis of mRNA levels of the parental control, deletion mutants and complemented strains of the four genes in the absence (−) and presence (+) of α-pheromone. C. Northern analysis of pheromone-induced expression of *CSH1* and *PBR1* in deletion mutants missing both the high consensus (strong) WPRE and the low consensus (lc) (weak) WPRE, lcWPRE, in the absence (−) and presence (+) of α-pheromone (α-ph). D. Western analysis of pheromone-induced expression of *CSH1* and *PBR1* in deletion mutants, as in panel C, using anti-GFP antibody.

*EAP1* encodes a glycosylphosphatidylinositol-anchored cell wall protein that functions as an adhesin in biofilm development in *S. cerevisiae* as well as in an **a**/α strain of *C. albicans* [35]–[37]. *PGA10*, also known as *RBT51*, encodes a putative hydrophobic extracellular membrane protein that plays a role in adhesion and biofilm development in an **a**/α strain of *C. albicans* [38]. In the complemented strains *EAP1_WPREΔ_-EAP1*/*eap1* and *PGA10_WPREΔ_-PGA10*/*pga10*, GFP-tagged Eap1 and GFP-tagged Pga10, respectively, localized at the surface of α-pheromone-induced white cells (Figure 2A). *CSH1* encodes a protein involved in cell surface hydrophobicity in **a**/α cells [39],[40] and *PBR1* will be shown here to play a role in biofilm development in *MTL*-homozygous cells. In the complemented controls *CSH1_WPREΔ_-CSH1*/*csh1* and *PBR1_WPREΔ_-PBR1*/*pbr1*, GFP-tagged Csh1 and GFP-tagged Pbr1, respectively, localized primarily in the cytoplasm of α-pheromone-induced white cells (Figure 2A).

To test whether α-pheromone activated the four white-specific genes through the putative pheromone response element *WPRE*, expression of each gene was compared by northern analysis to the parental strain P37005, the homozygous deletion mutant, the *WPRE* deletion mutant and the complemented control, in the absence and presence of α-pheromone.

### *EAP1*

In the absence of α-pheromone, *EAP1* was expressed at a basal level in white cells of the parental (P37005) strain, the WPRE deletion mutant and the complemented control strain (Figure 2B). α-pheromone up-regulated expression in both the parental and complemented control strains (Figure 2B). Expression in strain *EAP1*_WPREΔ_/*eap1* remained at the basal level in the absence or presence of α-pheromone (Figure 2B).

### *PGA10* and *CSH*

In the absence of α-pheromone, *PGA10* and *CSH1* were expressed at basal levels in white cells of the parental strain, the complemented control strains and the WPRE deletion mutants (Figure 2B). α-pheromone up-regulated expression of *PGA10* and *CSH1* in both the parental and complemented control strains by more than five- and six-fold, respectively (Figure 2B). α-pheromone also up-regulated expression of *PGA10* and *CSH1* in the WPRE deletion mutants, but to less than a third of the stimulated level in the parental or complemented control strains (Figure 2B).

### *PBR1*

In the absence of α-pheromone, *PBR1* expression was undetectable in the white cells of the parental strain, complemented control and the WPRE deletion mutant (Figure 2B). There appeared, therefore, to be no basal expression, as there was for the other three genes tested. α-pheromone up-regulated PBR1 expression in the parental and complemented control strain (Figure 2B). It also up-regulated *PBR1* expression in the WPRE deletion mutant, but to a level only one tenth that of stimulated parental and complemented control cells (Figure 2B).

The low but reproducible levels of expression of *PGA10*, *CSH1* and *PBR1* induced by α-pheromone in the respective WPRE deletion mutants could have been mediated by a second, weaker pheromone-response element in the promoters of each of the three genes. We identified a lower consensus WPRE (lcWPRE) in the promoter of each of the three genes *PGA10*, *CSH1* and *PBR1*, located between −432 and −418, −408 and −394, and −262 and −248 bp, respectively. Low consensus WPRE sequences in all 12 white-specific pheromone-induced genes are described in supplemental Table S5. To test whether these sites could be responsible for low level pheromone induction, we deleted them from strains *CSH1_WPREΔ_/csh1* and *PBR1_WPREΔ_/pbr1*, generating strains *CSH1_WPREΔlcWPREΔ_/csh1* and *PBR1_WPREΔlcWPREΔ_/pbr1*. Deletion of the lcWPRE in the promoters of both of the WPRE mutants completely eliminated low level induction by pheromone in white cells (Figure 2C). These results indicate that the lcWPREs in the promoters of *CSH1* and *PBR1* were responsible for the low level of residual induction by pheromone observed in the WPRE deletion mutants (Figure 2B). We also compared, by western analysis using antibody against the GFP tags, the levels of the proteins Csh1 and Pbr1 in the WPRE deletion mutants *CSH1_WPREΔ_/csh1* and *CSH1_WPREΔlcWPREΔ_/csh1*, and *PBR1_WPREΔ_/pbr1* and *PBR1_WPREΔlcWPREΔ_/pbr1*, respectively, with the complemented controls. The levels of proteins were remarkably consistent with the transcription levels (Figure 2D).

Finally, we found that as is the case for other white-specific genes [25],[26], up-regulation of the four selected genes *EAP1*, *PGA10*, *CSH1* and *PBR1* by pheromone was blocked in the mutants *ste4/ste4* and the double mutant *cek1/cek1 cek2/cek2*, but not in the mutant *cph1/cph1* (data not shown). These results demonstrate that as is the case for white-specific genes in general, up-regulation of these genes by pheromone depends upon the MAP kinase pathway, but not the target transcription factor Cph1.

### OPRE regulates pheromone-induced opaque-specific gene expression

**Figure 3:**
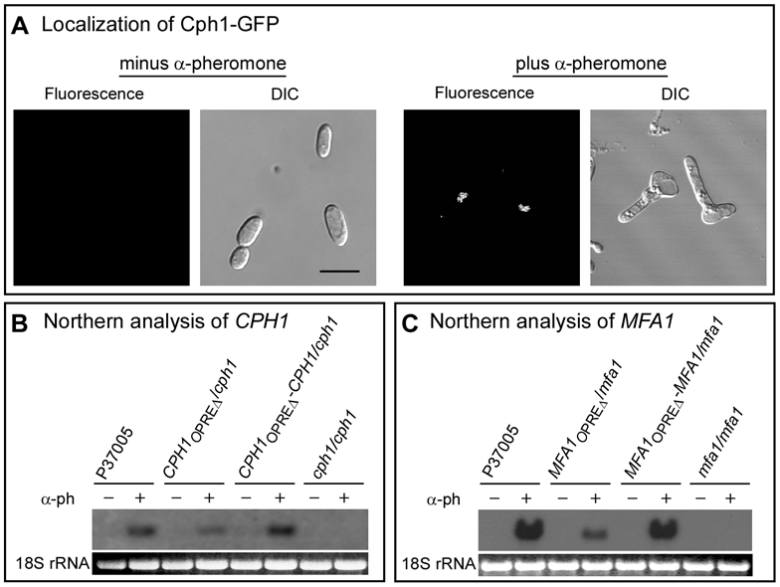
Up-regulation of the genes *CPH1* and *MFA1* by α-pheromone requires the opaque-specific pheromone response element OPRE. A. GFP visualization reveals that Cph1 localizes to the putative nucleus. The strain *CPH1*_OPREΔ_-*CPH1/cph1*, which possesses a C-terminal GFP tag, was used for analysis. B. Northern analysis of the RNA levels of the parental control, deletion mutants and complemented strains for *CPH1* in the absence (−) and presence (+) of α-pheromone (α-ph). C. Northern analysis of the RNA levels of the parental control, deletion mutants and complemented strains for *MFA1* in the absence (−) and presence (+) of α-pheromone (α-ph).

To assess whether the putative opaque pheromone response element OPRE mediated pheromone induction of opaque-specific genes, heterozygous deletion mutants were generated for *CPH1* and *MFA1*, genes selectively up-regulated by α-pheromone in opaque but not white cells [25]. *CPH1* encodes the gene for the downstream transcription factor that activates opaque-specific genes and *MFA1* encodes the gene for the **a**-pheromone. The OPRE in the promoter of the retained *CPH1* and *MFA1* copy of the heterozygote *CPH1/cph1* and *MFA1/mfa1*, respectively, were then selectively deleted, resulting in the OPRE deletion mutants *CPH1*_OPREΔ_/*cph1* and *MFA1*_OPREΔ_/*mfa1*. The *CPH1*_OPREΔ_ and the *MFA1*_OPREΔ_ copy in the respective mutants were then replaced with the wild type ORF and promoter to generate the complemented control *CPH1*_OPREΔ_-*CPH1*/*cph1* and *MFA1*_OPREΔ_-*MFA1*/*mfa1*. The GFP genes were fused in-frame for the localization and western studies. The wild type gene copy in both *CPH1/cph1* and *MFA1/mfa1* were also deleted to generate the homozygous deletion mutants *cph1/cph1* and *mfa1/mfa1*. The GFP-tagged Cph1 protein in strain *CPH1*_OPREΔ_-*CPH1/cph1* localized in the nucleus of α-pheromone-treated opaque cells of the complemented strain, as would be expected for an opaque-specific *trans*-acting factor (Figure 3A).

Expression of *CPH1* and *MFA1* was then assessed by northern analysis in the parental strain P37005, the homozygous deletion mutants, the OPREΔ mutants and the complemented controls, in the absence or presence of α-pheromone. In the absence of α-pheromone, *CPH1* and *MFA1* expression was undetectable in opaque cells of the parental strain, complemented control and the OPRE deletion mutants (Figure 3B, C). α-pheromone up-regulated expression of *CPH1* and *MFA1* in both the parental and complemented control strains (Figure 3B, C). It also up-regulated *CPH1* and *MFA1* in the OPRE deletion mutants, but to only approximately one tenth the level of parental or complemented control cells (Figure 3B, C). These low levels of activation could have been mediated by weaker OPREs, as was the case for WPRE mutants. A site with lower OPRE homology to the consensus sequence was identified in both the *CPH1* and *MFA1* promoters (supplemental Table S6). These results indicate that the OPRE, not the *S. cerevisiae* PRE-like sequences identified by Bennett and Johnson [27], function as the major response elements in the promoters of genes up-regulated by α-pheromone in opaque cells.

To provide further support to the suggestion that Cph1 up-regulates opaque-specific genes through the OPRE, we analyzed the expression of the genes *MFA1* and *KAR4*, which both contain an OPRE (supplemental Table S6), in the mutant *cph1/cph1*. Neither of these genes were up-regulated by α-pheromone in this mutant ([25]; S. Yi and D. R. Soll, unpublished observations). The results demonstrate that a cell must contain a functional Cph1 for α-pheromone-induced expression of genes regulated through OPRE.

### The four pheromone-induced white-specific genes play no role in the opaque pheromone response

**Figure 4:**
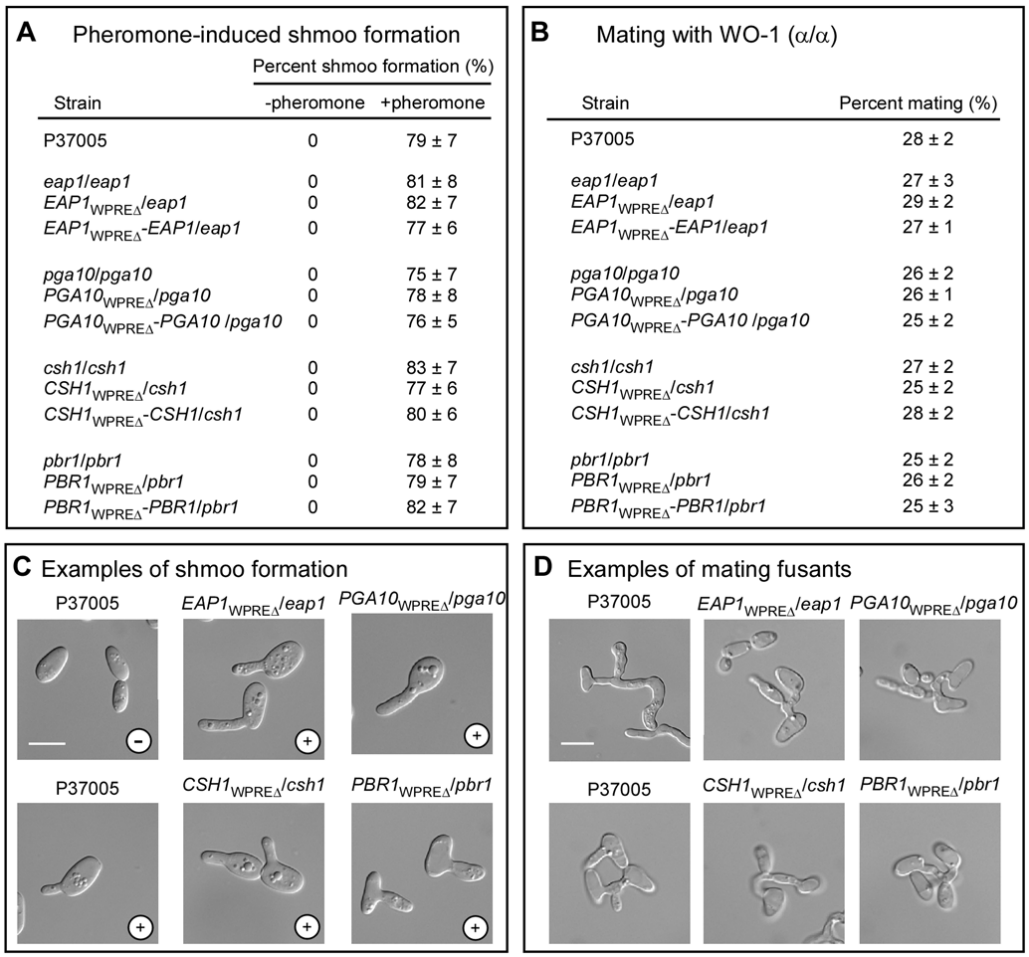
The genes *EAP1*, *PGA10*, *CSH1* and *PBR1* are not necessary for α-pheromone-induced shmoo formation or mating. A. Quantitation of shmoo formation of control and mutant strains in response to 4 hr treatment with 3×10^−6^ M α-pheromone (chemically synthesized 13-mer). At least 1,000 cells, the sum of four independent experiments, were analyzed and the mean±standard deviation of the percent shmoo formation presented. N.S., not significant. B. Quantitation of fusion between control and mutant opaque cells, with opaque α/α cells of the mating partner WO-1. At least 2,000 cells of each strain, the sum of four independent experiments, were analyzed and the mean±standard deviation of the percent presented. C. Examples of shmoo formation. D. Examples of mating fusants with α/α strain WO-1. −, absence of α-pheromone; +, presence of α-pheromone. Scale bars in C and D represent 4 µm.

Northern analysis revealed that nine of the twelve α-pheromone-induced, white-specific genes had no detectable signal in opaque cells in the absence or presence of α-pheromone, and three (*PHR1*, *CIT1*, *RBT5*) were expressed at the same basal levels in the absence and presence of α-pheromone (Figure 1A). One would assume, therefore, that none of these white-specific genes played a role in the opaque-cell response to pheromone. To directly test this assumption, we examined whether opaque cells of the homozygous and WPRE deletion mutants of the four genes *EAP1*, *PGA10*, *CSH1* and *PBR1*, formed shmoos in response to α-pheromone or mated with opaque cells of the natural α/α strain WO-1. Both the frequency of pheromone-induced shmoo formations (Figure 4A) and that of fusion in mating mixtures (Figure 4B) were indistinguishable among opaque cells of the parental strain P37005, the homozygous deletion mutants, the WPRE deletion mutants and the complemented control strains. The shmoos (Figure 4C) formed by opaque cells of the mutants in response to α-pheromone, and the mating fusions formed between opaque **a**/**a** mutant cells and opaque α/α WO-1 cells (Figure 4D), were indistinguishable from those of the parental strain P37005. These results indicate that pheromone-induced, white-specific genes do not play a role in the mating process of opaque cells.

### All four test genes play a role in the white cell adhesion response

**Figure 5:**
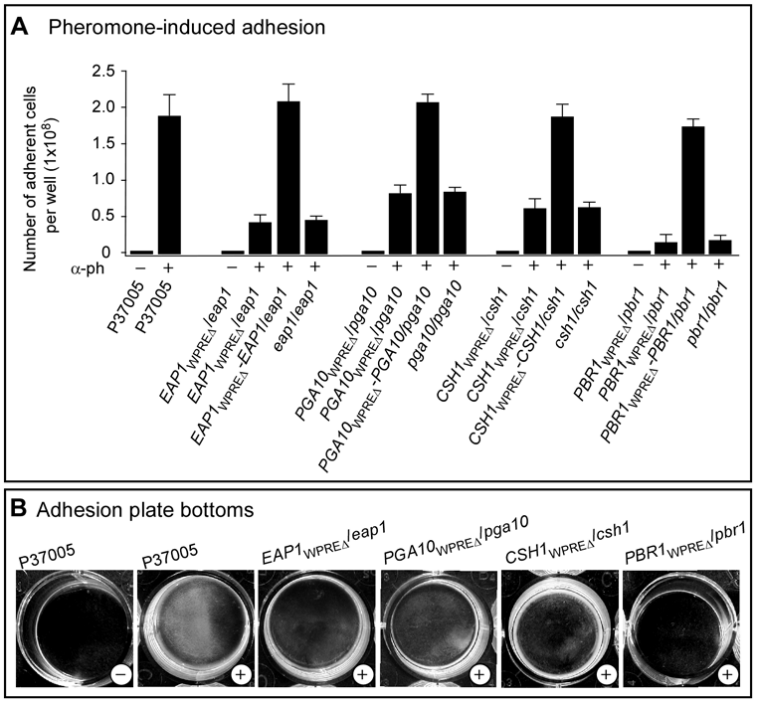
The genes *EAP1*, *PGA10*, *CSH1* and *PBR1* all play a role in α-pheromone-induced white cell adhesion. A. Quantitation of cells adhering to the well bottom in the absence (−) and presence (+) of α-pheromone (α-ph). The mean±standard deviation (error bars) of three dishes is presented. B. Examples of the bottom of wells after washing of control and mutant strains.

In response to pheromone, white cells undergo dramatic increases in cohesion, as well as adhesion to a substratum [20],[25],[26]. To test whether the four selected white-specific genes up-regulated by α-pheromone played a role in adhesion, we compared this pheromone response between parent and mutant strains. α-pheromone induced more than 90% of the white cell populations of parental strain P37005 to adhere to the bottom of a plastic dish (Figure 5A). This represented more than a 100 fold increase over untreated cells. Although α-pheromone also induced increases in adhesion in the four homozygous deletion mutants *eap1*/*eap1*, *pga10*/*pga10*, *csh1*/*csh1* and *pbr1*/*pbr1*, the induced levels were 24%, 43%, 38% and 14%, respectively, that of the parental strain (Figure 5A). Deletion of just the WPRE region with the highest consensus in the promoter of each of the four genes resulted in approximately the same reductions in pheromone-induced adhesion as the homozygous deletion mutants (Figure 5A). Examples of the densities of cells adhering to the dish bottoms in the parent and WPRE deletion mutants are presented in Figure 5B. These results demonstrate that all four genes played a role in pheromone-induced adhesion, but no single gene was sufficient for the full pheromone-induced response. It should be noted that the lowest level of α-pheromone-induced adhesion was obtained in the homozygous and WPRE deletion mutants of *PBR1* (Figure 5A). It should also be noted that complementation of each WPRE deletion mutant with the native gene resulted in the reestablishment of wild type-level adhesion (Figure 5A).

Since the low level induction by α-pheromone of *CSH1* and *PBR1* in the WPRE deletion mutants was eliminated by deletion of a second, lower consensus WPRE (Figure 2C), lcWPRE, we tested whether there was a further reduction in pheromone-induced adhesion in the deletion mutants *CSH1*_WPREΔlcWPREΔ_/*csh1* and *PBR1*_WPREΔlcWPREΔ_/*pbr1*. The levels of adhesion were the same as in the mutants *CSH1*_WPREΔ_/*csh1* and *PBR1*_WPREΔ_/*pbr1* (supplemental Figure S2), indicating that the induced residual levels of adhesion in these mutants were due to the action of other gene products.

### All four genes play a role in the white cell biofilm response

In the absence of opaque cells, biofilm formation by white cells is dependent upon a functional white cell pheromone response pathway [26]. Such white cell biofilms are enhanced by adding as little as 1% opaque cells [20],[25]. We analyzed the role of the four pheromone-induced white-specific genes on white cell biofilm formation in the absence of opaque cells and in the presence of 10% opaque cells, the latter containing a 1∶1 ratio of opaque **a**/**a** and opaque α/α cells. Presumably the opaque **a**/**a** cells, through the release of **a**-pheromone, up-regulate α-pheromone synthesis in the opaque α/α cells, providing a continuous source of α-pheromone for majority white **a**/**a** cell stimulation [20]. Biofilms were cast on a silicone elastomer surface, incubated for 48 hour, fixed and analyzed for the formation of a basal layer of white cells, hyphae formation in the upper region of the biofilm, hypha orientation, matrix formation and biofilm thickness, using laser scanning confocal microscopy.

**Figure 6:**
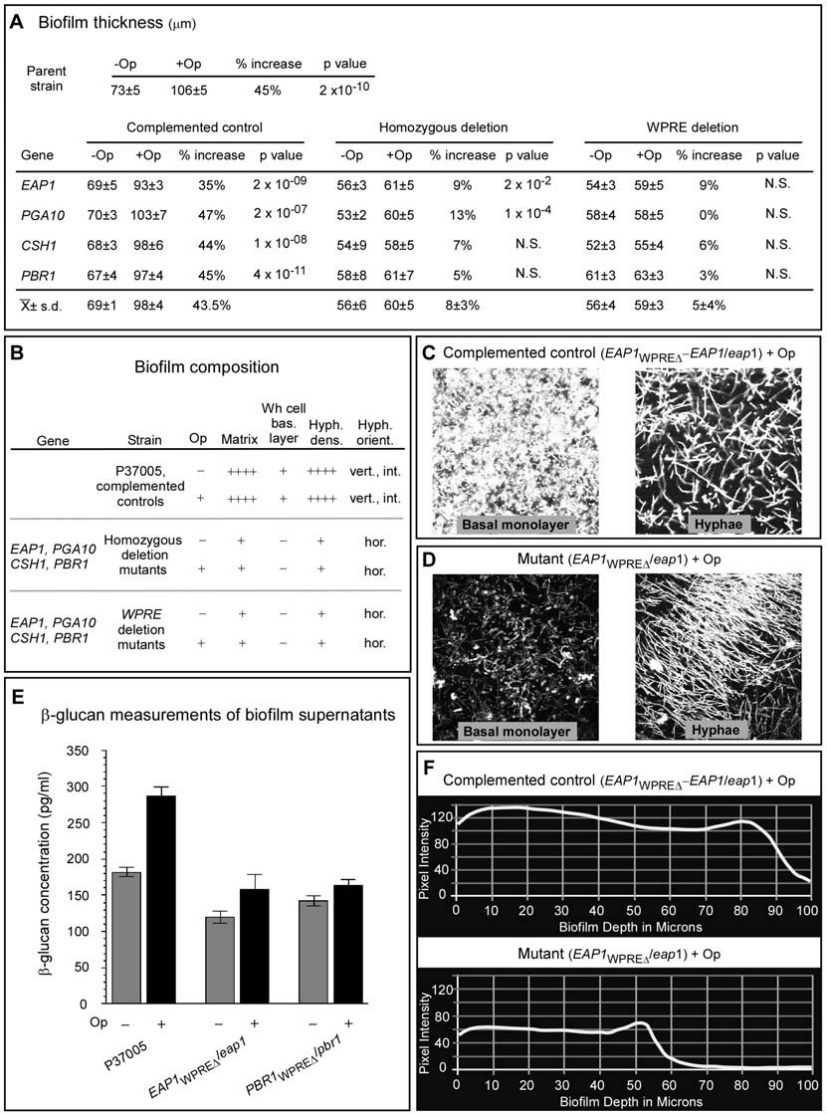
The genes *EAP1*, *PGA10*, *CSH1* and *PBR1* all are necessary for white a/a cell biofilm development in the absence or presence of minority opaque cells. A. Biofilm thickness measured in µm in the absence (−Op) and presence (+Op) of 10% opaque cells, for the complemented control strains, homozygous deletion strains and WPRE deletion strains of the four test genes. The opaque cells were half a/a and α/α. For each strain and condition, three individual biofilms were analyzed through three random regions, providing nine measurements. P values are provided for the measurements in the absence (−) and presence (+) of 10% opaque cells (Op). In supplemental Table S7, the p values are presented for comparisons of the complemented control and the two deletion mutants. B. Comparisons of biofilm compositions of the parent strain P37005, the complemented control strains, the homozygous mutants and the WPRE deletion mutants of the four test genes, in the absence (−) or presence (+) of opaque (Op) cells. Maximum matrix staining is representative as ++++ and minimum as +. The presence or absence of a white cell basal layer (Wh cell bas. layer) is denoted as + or −, respectively. Maximum and minimum hyphal density (Hyph. des) is represented as ++++ and +, respectively. Hyphal orientation (Hyph. orient.) was either vertical (vert.) and intertwined (int.), or horizontal (hor.). C, D. Scanning confocal microscopic images of the basal layer and hyphal region of biofilms of the *EAP1* complemented control and WPRE deletion mutant of *EAP1*. E. β-glucan measurements of biofilm supernatants F. Examples of the pixel intensity scans used to measure thickness, for the complemented control and WPRE deletion mutant of *EAP1*, respectively.

The mean thickness of biofilms formed by majority white cells in the absence of minority opaque cells of the parent strain was 73±5 µm; that of the four complemented control strains averaged 69±1 µm (Figure 6A). The differences were not significantly different (the p values were greater than 0.05). In the presence of minority opaque cells, the mean thickness of the biofilms formed by the parent strain P37005 was 106±5 µm and the average of the four complemented strains 98±4 µm, which represented increases in thickness of 45% and 43%, respectively, over that in the absence of opaque cells (Figure 6A). The differences in the absence and presence of minority opaque cells were significant (Figure 6A). In both the absence and presence of minority opaque cells, the biofilms formed by white cells of the parent and complemented control strains possessed a basal layer of white cells, and above this layer a region of intertwined hyphae oriented vertically (Figure 6B, C). These biofilms contained an extracellular matrix that stained with calcofluor (supplemental Figure S1A). Measurements were also made of the concentration of β-glucan in the supernatant of biofilm cultures of the parental strain P37005 in the absence and presence of minority opaque cells [41]. The concentration was 58% higher in the presence of opaque cells than it was in the absence (Figure 6E). The difference was significant.

In the absence of opaque cells, the thickness of the biofilms of the four homozygous deletion mutants averaged 56±6 µm and that of the four WPRE deletion mutants 56±4 µm (Figure 6A). These biofilms were, therefore, on average 20% thinner than those of parental and complemented control cells (Figure 6A). These differences were significant (supplemental Table S7). The presence of opaque cells had only a marginal effect on the thickness of the biofilm formed by the deletion mutants (Figure 6A). Examples of the pixel intensity scans used to measure thickness for control and mutant cell biofilms are presented in Figure 6F.

In both the absence and presence of opaque cells, the biofilms formed by the deletion mutants had no consistent white cell basal layer; the cells at the substratum were sparse or patchy (Figure 6B, D). The matrix also strained far less intensely than that of control strains (Figure 6B and supplemental Figure S1B). In addition, the hyphae formed as patches and were orientated horizontally (*i.e.*, in parallel with the substratum) (Figure 6B, D), rather than vertically, in contrast to the vertical orientation in control cell biofilms (Figure 6B, C). This aberrant orientation may have been due to the dramatic decrease in matrix suggested by the staining results (supplemental Figure S1). Measurements of β-glucan in the supernatant of biofilms revealed significant differences between mutants and the parental stain both in the absence and presence of minority opaque cells. For the WPRE deletion mutants *EAP1_WPREΔ_/eap1* and *PBR1_WPREΔ_/pbr1*, the levels of β-glucan were on average 33% and 19% lower, respectively, in the absence of opaque cells, and 44% and 42% lower, respectively, in the presence of opaque cells (Figure 6E). These differences proved significant. Together these results demonstrate that each of the four α-pheromone-induced, white-specific genes analyzed was essential for normal biofilm formation and architecture in the absence as well as in the presence of opaque cells.

### Expression patterns in deletion mutants of components of the pheromone response pathway

**Figure 7:**
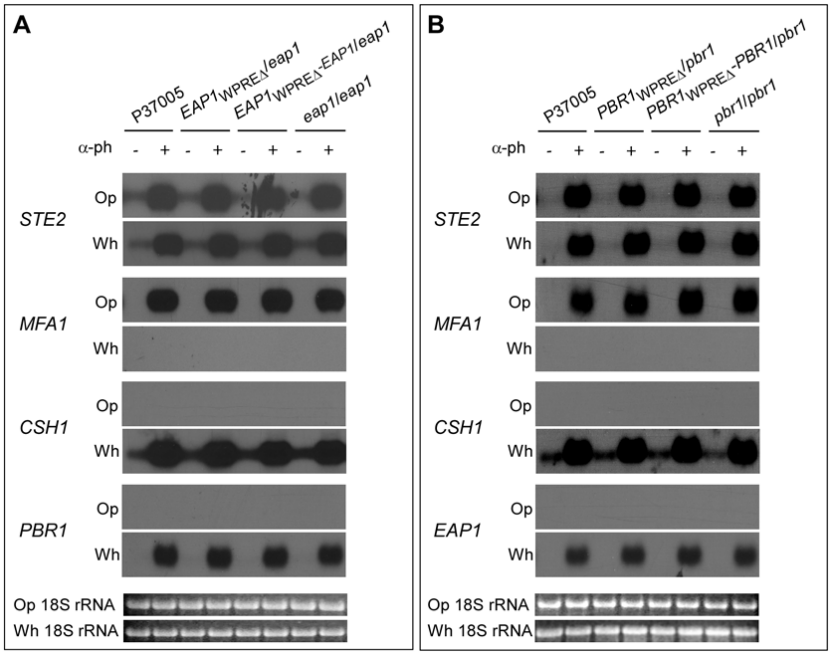
Deletion of *EAP1* or *PBR1* has no effect on pheromone regulation of *STE2*, *MFA1*, *CSH1*, and *EAP1* or *PBR1* expression, as demonstrated by northern blot hybridization. A. Expression of the four genes in *EAP1* mutants in the absence (−) or presence (+) of α-pheromone. B. Expression of the four genes in *PBR1* mutants in the absence (−) or presence (+) of α-pheromone. To demonstrate levels of loading, 18S rRNA levels are shown for opaque and white.

Although the four WPRE-regulated white-specific genes are activated by a downstream transcription factor that is induced by the pheromone-activated MAP kinase pathway [25],[26], this does not exclude them from playing a role in regulating upstream genes in the pheromone response pathway by a loop-back control mechanism. We, therefore, tested whether pheromone up-regulated the α-pheromone receptor gene, *STE2* and the mating factor **a** gene, *MFA1*, in the homozygous and WPRE deletion mutants of *EAP1* and *PBR1*. We also tested whether *CSH1* and *PBR1* were up-regulated by α-pheromone in the *EAP1* deletion mutants, and whether *CSH1* and *EAP1* were up-regulated in the *PBR1* deletion mutants. No effects were observed on expression (Figure 7A, B). Similar results were obtained for the homozygous and WPRE deletion mutants of *CSH1* (data not shown). These results indicate that pheromone-induced, white specific genes involved in adhesion and biofilm development do not play a role in the transduction of the pheromone signal or in up-regulation of other pheromone induced genes.

### Overexpressing *PBR1* in the other deletion mutants

**Figure 8:**
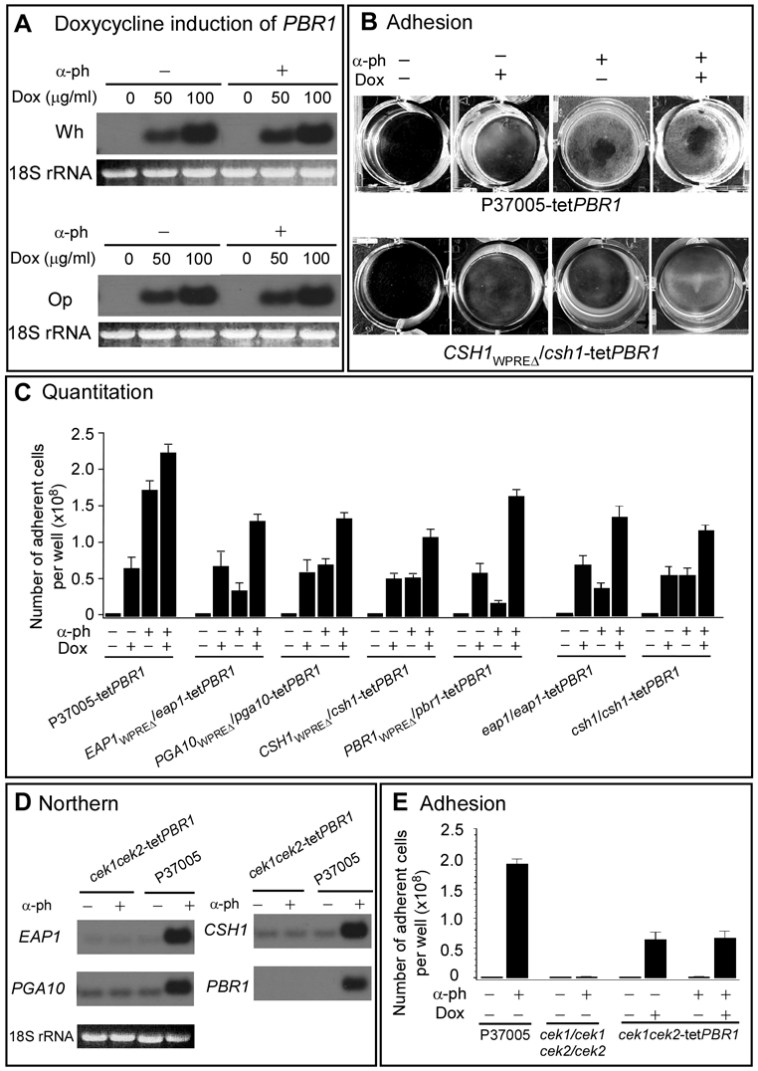
Overexpression of *PBR1* at the ectopic locus *ADH1* in the parental strain and in WPRE deletion mutants of *EAP1*, *PGA10*, *CSH1* and *PBR1*, induces partial adhesion or enhances adhesion in the absence of α-pheromone and in the absence of *EAP1*, *PGA10* and *CSH1* expression. A. Northern analysis demonstrating that 100 µg/ml of doxycycline (Dox) induces *PBR1* transcription similarly in the absence (−) and presence (+) of α-pheromone (α-ph). B. Examples of white cells adhering to the bottom of wells for the control strain P37005-tet*PBR1* and *CSH1* WPRE deletion mutant *CSH1_WPREΔ_/csh1*-tet*PBR1* in the absence (−) or presence (+) of doxycycline. C. Quantitation of adherence to well bottoms. D. Northern analysis demonstrating treatment with pheromone of the mutant *cek1/cek1 cek2/cek2* does not cause an increase in expression of the four test genes necessary for a full adhesion response to α-pheromone. E. Demonstration that misexpression of *PBR1* in the mutant *cek1/cek1 cek2/cek2*, in which the three genes, *EAP1*, *PGA10*, *CSH1* and the native *PBR1* gene are not up-regulated, results in an increase in adhesion that is 33% that of control cells. This increase is therefore independent of α-pheromone treatment in the *cek1/cek1 cek2/cek2* mutant.

Homozygous and WPRE deletion mutants of the four white-specific genes exhibited large but incomplete reductions in adhesion (Figure 5A). The largest effect was by the homozygous and WPRE deletion mutants of *PBR1* (Figure 5A, B). These results suggested that the contributions of the four tested genes to the adhesion response may be both independent and additive. To explore this hypothesis, we transformed the parent strain P37005, and the WPRE deletion mutants *EAP1_WPREΔ_/eap1*, *PGA10_WPREΔ_/pga10*, *CSH1_WPREΔ_/csh1* and *PBR1_WPREΔ_/pbr1* with a construct in which *PBR1* was under the control of the inducible *tetracycline* promoter [42]. The construct was targeted to one of the two alleles of the *ADH1* gene [42]. The resulting strains were P37005-tet*PBR1* (the control), *EAP1_WPREΔ_/eap1-tetPBR1*, *PGA10_WPREΔ_/pga10-tetPBR1*, *CSH1_WPREΔ_/csh1-tetPBR1* and *PBR1_WPREΔ_/pbr1-tetPBR1*. Up-regulation of the tetracycline regulated gene by the tetracycline analog doxycycline was demonstrated to be dose-dependent and independent of pheromone, as demonstrated in *PBR1_WPREΔ_/pbr1-tetPBR1* (Figure 8A).

Misexpression of *PBR1* in the absence of pheromone caused an increase in adhesion in the transformed parental strain P37005 that was approximately one third of the increase induced by α-pheromone (Figure 8B, C). Misexpression in the four transformed WPRE deletion mutants in the absence of α-pheromone resulted in similar levels of induction (Figure 8B, C). In the presence of α-pheromone, *PBR1* misexpression in both the transformed parental strain and the four WPRE deletion mutants resulted in a level of adhesion greater than when *PBR1* was misexpressed in the absence of α-pheromone or when cells were only treated with α-pheromone (*i.e.*, in the absence of doxycycline) (Figure 8B, C). These results indicated that the expression of *PBR1* in the absence of pheromone-induced expression of the other three test genes resulted in increased adhesion, and that simultaneous *PBR1* misexpression and α-pheromone induced native gene expression had an additive effect on adhesion. To explore this point further, we transformed the double mutant *cek1/cek1 cek2/cek2* [25] with the misexpression module at the *ADH1* locus. This mutant did not undergo α-pheromone induction of *EAP1*, *PGA10*, *CSH1* or native *PBR1* (Figure 8D). Misexpression of *PBR1* in the double mutant in the absence or presence of α-pheromone resulted in an increase in adhesion to a level again one third of the α-pheromone-induced level in the parental control (Figure 8E). This result supports the suggestion that expression of *PBR1* alone results in increased adhesion, but not to control levels.

Since the WPRE deletion mutants of *EAP1*, *PGA10* and *CSH1* express these genes at basal or slightly induced levels (Figure 2B), misexpression of *PBR1* in these WPRE deletion mutants might still result in interactions between Pbr1 and the gene products Eap1, Pga10 or Csh1, respectively. To test further for independence, the deletion mutants *eap1/eap1* and *csh1/csh1* were transformed with the vector containing tet*PBR1* to generate *eap1/eap1*-tet*PBR1* and *csh1/csh1*-tet*PBR1*, and adhesion assessed in the absence or presence of α-pheromone and/or doxycycline. The results were highly similar to those obtained with the WPRE mutants transformed with tet*PBR1* (Figure 8C). These results support the suggestion that the white-specific α-pheromone-induced genes may confer adhesion independently and additively.

## Discussion

### Differences in the opaque and white response pathways

**Figure 9:**
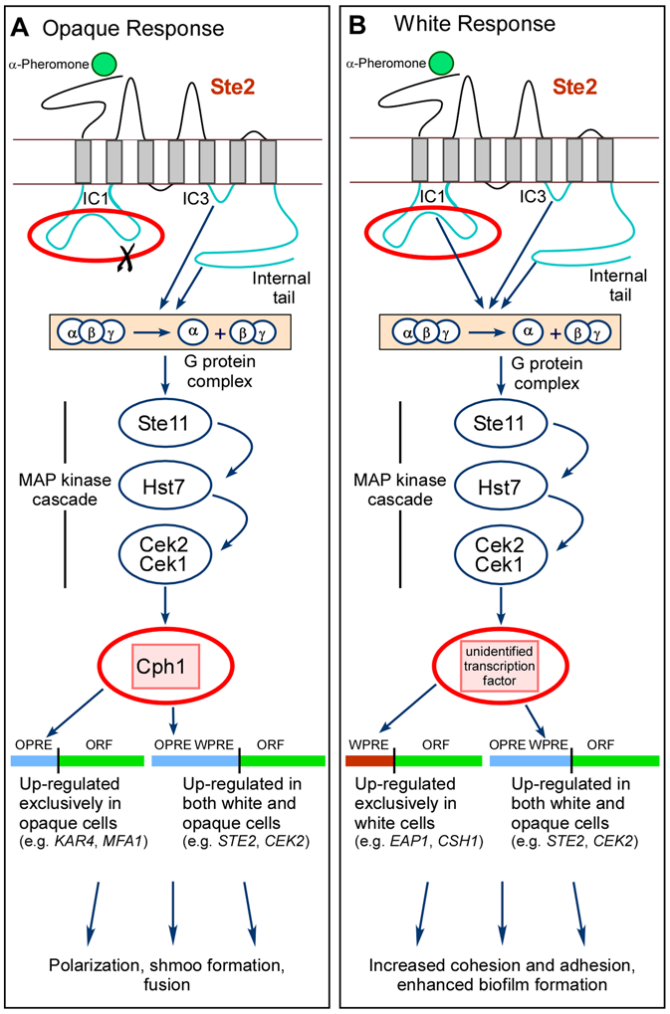
An updated model of the pathways regulating the pheromone-induced opaque (A) and white (B) responses that includes the downstream genes that are up-regulated through the opaque- and white-specific pheromone-response elements OPRE (A) and WPRE (B). This model also includes genes that are up-regulated in both the opaque and white responses, and therefore contain both an OPRE and a WPRE in their promoters. The two pathways share the same components from receptor through the MAP kinase cascade [25]. However, there are two differences, circled in red. First, an extra region of the first intracellular loop (ICI) is essential for the white, but not the opaque response [26]. Second, the transcription factor targeted by the pathway is *CPH1* in the opaque response and a still unidentified factor in the white response [25].

The α-pheromone response pathway of white cells, from receptor through the MAP kinase cascade, includes the same gene products as the pheromone response pathway of opaque cells (Figure 9) [25],[26]. However, the downstream targets of the pathways differ. The target of the opaque pathway is the transcription factor Cph1, a homolog of the *S. cerevisiae* transcription factor Ste12 [43]–[45]. The target of the white pathway, however, is a transcription factor that is distinct from Cph1 and remains unidentified [25]. In *S. cerevisiae*, pheromone up-regulates genes involved in the mating response through the transcription factor Ste12, which binds to a common pheromone response element, PRE [31]–[33]. Here we have presented evidence that in opaque cells, Cph1 activates opaque-specific genes by binding to a GC-rich, opaque-specific pheromone response element, OPRE, not a *S. cerevisiae* PRE-like sequence, as has been suggested [27]. We have also presented evidence that in white cells, the white-specific transcription factor activates white-specific genes by binding to an AT-rich, white-specific pheromone response element, WPRE (Figure 9). The promoter of each opaque-specific gene contains at least one OPRE and no WPRE, and the promoter of each white-specific gene contains at least one WPRE and no OPRE. Genes up-regulated by pheromone in both white and opaque cells have both a WPRE and an OPRE (Figure 9). The strategy for gene regulation in the white pheromone response therefore appears to involve a single white-specific transcription factor and a single white-specific *cis*-acting promoter element, and the strategy for gene regulation in the opaque pheromone response appears to involve a single opaque-specific transcription factor and a single opaque-specific *cis*-acting promoter element (Figure 9). These strategies are highly similar to that of the homologous mating pathway in *S. cerevisiae*, which also activates genes through one primary transcription factor and a single, dominant *cis*-acting regulatory element [31]–[33],[44],[45].

### White-specific genes and the adhesion response

Each of the twelve genes that we found were strongly up-regulated by pheromone in white but not opaque cells, contained a WPRE. Deletion of four of these genes or deletion of the WPRE from their promoters resulted in a marked reduction in the white cell adhesion response to pheromone. But in no case did the full deletion of the gene, the WPRE of the representative gene, or the WPRE and lcWPRE of the representative gene in combination, lead to the complete loss of the adhesion response, suggesting that the protein product of each gene may make an independent but additive contribution to the pheromone adhesion response. Hence, none of the four gene products appeared to be essential for the entirety of the adhesion response to pheromone. The experiments performed in which *PBR1* was misexpressed revealed that increased expression of *PBR1* alone, in the complete absence of expression of either *EAP1* or *CSH1* (*i.e.*, in the null mutants), resulted in a further, but incomplete, increase in adhesion. This was true for the WPRE and full homozygous deletion mutants as well. Moreover, misexpression of *PBR1* in the mutant *cek1/cek1 cek2/cek2*, in which *EAP1*, *PGA10*, *CSH1* and native *PBR1* cannot be induced, results in an increase in adhesion to 34% that of the induced control strain. These results indicate that the adhesion response contributed by *PBR1* in white cells may be independent of the adhesion response contributed by the other three genes. Hence, α-pheromone induced adhesion in white cells may represent the sum of multiple adhesion systems.

### White-specific genes and the biofilm response

Deletion of each one of the four randomly selected white-specific genes resulted in a reproducible loss in the adhesion response to α-pheromone, ranging between 57 and 86%. In contrast, deleting any one of the four genes or their WPREs resulted in relatively uniform defects in biofilm formation. Since biofilm formation is a complex process involving multiple cell phenotypes, matrix formation and a temporal sequence of steps during maturation [46]–[53], one might have expected that deletion of each of the four genes would cause a different and partial defect. If, however, all four genes played roles in an early step in biofilm development, such as the formation of the initial basal layer of cells on the substratum [50],[54],[55], then all subsequent steps might be similarly defective in the four deletion mutants, thus accounting for the uniformity of the biofilm defects.

### Induction of white cell biofilm formation in the absence of minority opaque cells

Perhaps our most surprising observation was that the major defects in biofilm development by white **a**/**a** cells were exhibited by the homozygous and WPRE deletion mutants of the four white-specific genes in the absence as well as in the presence of minority opaque cells. The defects included the absence of a continuous, dense basal layer on a silicone elastomer substratum, horizontally oriented patches of hyphae rather than uniformly dense, vertically intertwined hyphae in the domains above the basal layer, and a dramatic reduction of the extracellular matrix. These defects occurred in the absence as well as the presence of minority opaque cells, indicating that white cells possess the ability to autoactivate. Given that the white response, which includes increased adhesion and up-regulation of white specific genes, depends upon an exogenous source of pheromone, these results suggest one of two mechanisms. First, there may exist an autocrine system in which *MTL*-homozygous white cells, when placed on a surface under conditions conducive to biofilm formation, are able to self-stimulate by releasing pheromone of opposite mating type. This pheromone then binds to receptors on the same cells, activating the white cell response pathway. Such a scenario is plausible since white **a**/**a** cells possess the gene for α-pheromone and α/α cells possess the gene for the **a** pheromone. We had previously suggested that an autocrine system might regulate white cell biofilm formation based on the defective phenotype of the α-receptor deletion mutant, *ste2/ste2* [26]. The mutant formed a patchy biofilm with diminished thickness in the absence of minority opaque cells, similar to that observed for the mutants *eap1/eap1*, *pga10/pga10*, *csh1/csh1* and *pbr1/pbr1*. In a mechanism alternative to an autocrine system, white cells may depend upon the basal activity of the pheromone response pathway in the absence of pheromone, for normal biofilm formation. In *S. cerevisiae*, such basal activity has not only been observed in the pheromone response pathway [33], [56]–[58], but the **α** subunit of the trimeric G protein complex, *Gpα1*, which, when overexpressed, can activate changes in the mating response in the absence of pheromone [57]. Experiments are now in progress to distinguish between these alternatives.

### *MTL*-homozygous and *MTL*-heterozygous biofilms

Here, we have focused entirely on *MTL*-homozygous biofilm formation. *MTL*-heterozygous (**a**/α) strains, however, represent approximately 90% of natural strains [1]–[4] and hence must account for the majority of biofilms formed in nature. Interestingly, the majority of the twelve genes that were identified as strongly up-regulated by α-pheromone in white, but not opaque, **a**/**a** cells have been implicated directly or indirectly in the formation of **a**/α biofilms. In **a**/α cells, it has been demonstrated that 1) deletion of *EAP1* results in a decrease in binding to epithelial cells [59], and defective biofilm formation both in an *in vitro* parallel plate flow chamber model and in catheters [35]; 2) deletion of *PGA10* (*RBT5*) results in fragile biofilms [38]; 3) deletion of *CSH1* causes a reduction in hydrophilicity [39], a characteristic that enhances biofilm formation [60]; 4) *PHR1* is down-regulated in *sun41/sun41* mutants [61]; 5) and *LSP1* and *CIT1* are up-regulated during biofilm formation [62]. It is therefore imperative that biofilm formation by white *MTL*-homozygous cells and *MTL*-heterozygous cells be compared both at the morphological and molecular levels, that the regulation of the genes involved in **a**/α biofilm formation be elucidated and that the roles of the two different biofilms in pathogenesis be assessed.

### Evolution of the white cell pheromone response

It seems reasonable to hypothesize that the white cell biofilm response to pheromone evolved from the opaque cell mating response in *C. albicans*. The opaque response pathway is highly similar to that of *S. cerevisiae*, which branched from the *Candida* group early in the phylogenetic tree of the hemiascomycetes [63],[64]. Remarkably, neither *S. cerevisiae* nor members of the *Candida* group, other than *C. albicans* and the highly related species *Candida dubliniensis* [65],[66], undergo the white-opaque transition. The white response to pheromone appears to function solely to facilitate opaque cell mating through the genesis of a protective biofilm [20], suggesting that it arose from the opaque response in order to facilitate it [20]. Because the white cell pheromone response appears to be present only in *C. albicans*, it represents a pathway that has only recently evolved, and that appears to have borrowed the entire upper portion of the pheromone response pathway that functions in the mating process. The observations that the genes selectively up-regulated by α-pheromone in white, but not opaque, cells appear to play roles or to be regulated in the formation of **a**/α biofilms, also suggests that the target genes regulated by the white cell pheromone response pathway that are involved in white cell biofilm formation may have been derived from an ancestral program for **a**/α biofilm formation. Because it represents a recent event, the borrowed portions of the pathway appear to have had insufficient time to undergo refinement through gene replacement or alteration.

## Materials and Methods

### Strains and strain maintenance

Strain P37005, a natural clinical isolate from a blood stream infection with the *MTL* genotype **a**/**a** [1], was used to derive the homozygous deletion mutants and the WPRE mutants. The WPRE mutants were used to generate the complemented strains. All strains were maintained on agar containing modified Lee's medium [67],[68] supplemented with phloxine B, which differentially stains opaque sectors and colonies red [69].

### MEME identification of WPRE and OPRE

To identify candidate white- and opaque-specific *cis*-acting pheromone response elements (PREs) in *C. albicans*, a set of white phase-specific and opaque phase-specific pheromone-inducible genes were submitted to the motif-finding program, MEME [28]–[30]. All the selected genes were verified by northern analysis for induction by pheromone prior to motif analysis. One thousand base pairs upstream of the open reading frame of 12 white-specific and the 6 opaque-specific pheromone-up-regulated genes were pooled into two respective groups and subjected to MEME analysis. The website address for MEME is http://meme.sdsc.edu/meme/cgi-bin/meme.cgi. MEME program analyzes input promoter sequences for similarities among them and elicits consensus motifs, which may be present in some or all of the promoters analyzed. The parameters were set as follows: width of each motif between 6 and 15, a limit output of 3 different motif types, and other parameters with default values defined in the program. Results from the MEME analysis yields an E value defined as the probability of finding an equally well conserved pattern in random sequences [30]. The highest scoring hit in the analysis output for the white and opaque groups of genes represented the conserved DNA motifs, WPRE and OPRE, respectively. The WPRE or OPRE site was highlighted in each promoter and the distance from the translation start site shown.

To identify a strong WPRE or OPRE motif in the promoter, the parameter of “number of occurrences of a single motif” was set to be zero or one per sequence, and the result of MEME yielded one motif per promoter with the highest E value. To identify additional weaker WPRE or OPRE motifs in the promoter, the parameter of “number of occurrences of a single motif” was set to be any number of repetitions, and the result of MEME yielded more motifs, if any, per promoter with the same consensus but with lower E values. Noteworthy, the *S. cerevisiae* PRE (TGAAACA) [31]–[33] was not identified in our analysis with the same parameters, although a motif that contained a similar sequence to *S. cerevisiae* PRE was obtained with a poor E value of 10^+2^. The result is consistent with the observation by Bennett and Johnson [27], who reported that *S. cerevisiae* PRE was present in some, but not all, of genes that are induced by pheromone in opaque cells.

### Construction of WPRE mutants, WPRE-complemented strains and gene disruption mutants

The recyclable SAT1 flipper cassette SAT1-2A, containing a dominant nourseothricin resistance marker SAT^r^ [70], was used for all mutant construction according to a protocol previously described [25]. The original SAT1 flipper plasmid pSFS2A was a generous gift from Dr. Joachim Morschhäuser, the University of Würzburg, Germany. To generate WPRE mutants, a two-step strategy was employed. First, heterozygous mutants of the coding region were derived. In brief, 5′ and 3′ flanking regions of each gene were amplified by PCR using the primer pairs f1, r1, and f2, r2, respectively (supplemental Table S2). The 5′ and 3′ fragments were then each digested with SmaI and ligated together using T4 DNA ligase. The fusion product was amplified by PCR and cloned into pGEM-T Easy vector (Promega, Madison, WI), generating the plasmid pGeneX1-T. The SAT1-2A cassette was then inserted into the SmaI-digested, dephosphorylated plasmid pGeneX1-T, yielding pGeneX1-2A. This plasmid was digested with SacI and SacII (or PvuII in the case of *CSH1*), then introduced into *C. albicans* strain P37005 by electroporation [71]. The derived heterozygotes were confirmed by PCR and Southern analysis. The heterozygotes were subjected to a pop-out protocol in YPM medium [25] to excise the *CaSAT1* marker prior to the next step. Second, WPRE elements were deleted directly from the endogenous promoters in the heterozygous background. The methods used were as follows. A 5′ flanking DNA fragment, about 2 kb upstream of the start codon plus the ORF, was amplified using the primer pair pf1 and pr1 (supplemental Table S2). In addition, two DNA fragments spanning a promoter region on the 5′ and 3′ side, respectively, of the WPRE element to be deleted, were amplified by PCR with the primer pairs listed in Table S2. These two fragments, bordering the targeted WPRE element, were then fused by PCR using the primer pair pf2 and pr2 (supplemental Table S2), generating the 3′ flanking DNA fragment. The 5′ and 3′ flanking DNA fragment were then each digested with SmaI, fused together with T4 DNA ligase, amplified by PCR using primers pf1 and pr2 (supplemental ), and ligated into pGEM-T Easy vector (Promega, Madison, WI), generating the plasmid pGeneXw-T. The SAT1-2A cassette was inserted into the SmaI-digested, dephosphorylated plasmid pGeneXw-T, yielding pGeneXw-2A. This plasmid was digested with SacI and SacII (or PvuII in the case of *PGA10*), then introduced by electroporation [71] into the heterozygous strains obtained in the first step. Two or more independent WPRE deletion mutants were obtained for each gene and verified by PCR sequencing and Southern analysis. We generated in this way the mutants *EAP1_WPREΔ_/eap1*, *PGA10_WPREΔ_/pga10*, *CSH1_WPREΔ_/csh1*, and *PBR1_WPREΔ_/pbr1* (supplemental Table S4).

To obtain a complemented strain for each of the above WPRE deletion mutants, a DNA sequence containing an intact promoter region, the ORF and a C-terminal GFP fusion was designed to target the gene copy of each WPRE deletion mutant in which the WPRE was deleted. The SAT^r^ marker was first excised from the WPRE mutants in the medium YPM [25]. The 5′ region spanning the promoter and ORF was amplified by PCR with the primers wQ1f and wQ1r (supplemental Table S2). The 3′ region spanning a region downstream of the stop codon was amplified using the primers wQ2f and wQ2r (supplemental Table S2). The 5′-3′ fusion product was amplified by PCR and subcloned into pGEM-T Easy to derive pGeneXwQ-T. A fragment containing both *GFP* and the SAT^r^ marker was amplified by PCR with primers GFBhF1 and SATBgF1 (supplemental Table S2), using plasmid pK91.6 (T. Srikantha and D. R. Soll, unpublished) as template. This *GFP*-*SAT* fragment was digested with BamHI plus Bg1II and ligated into the BamHI (or BglII in the case of *CSH1*)-digested, dephosphorylated plasmid pGeneXwQ-T to derive plasmid pGeneXwQ-SAT. C-terminal GFP fusion was confirmed to be in-frame by sequencing. pGeneXwQ-SAT was then digested with SacI plus SacII and transformed into the WPRE deletion mutants. The resulting complemented strains were verified by PCR, sequencing and Southern analysis. We generated in this way the complemented control strains *EAP1_WPREΔ_-EAP1/eap1*, *PGA10_WPREΔ_-PGA10/pga10*, *CSH1_WPREΔ_-CSH1/csh1*, and *PBR1_WPREΔ_-PBR1/pbr1*.

Null mutants of each gene were also created. As described earlier, the heterozygous mutants were derived based on the selection marker SAT^r^ [70]. The deletion cassette for the second allele was then constructed using the same strategy to delete the first copy. The resulting plasmid pGeneX2-2A was digested with SacI and SacII, and transformed by electroporation into the heterozygous mutant strains for each gene. At least two independent null mutants for each gene were generated and confirmed by PCR and Southern analysis. We generated in this way the null mutants *eap1*/*eap1*, *pga10*/*pga10*, *csh1*/*csh1* and *pbr1*/*pbr1*.

### Northern analysis

The methods for northern blot hybridization have been described in detail [12],[25]. Probes were made by polymerase chain reaction (PCR) for genes that have been implicated in adhesion, cell wall biogenesis, biofilm formation and filamentation. The primers for synthesizing the probes for these genes are presented in supplemental Table S3. Quantitation for the signal intensity of each band in northern blots was performed by gray value analysis in the graphics program Adobe Photoshop™.

### Imaging GFP-tagged proteins

Fluorescence of GFP-tagged proteins was visualized through a ZEISS Axioplan2 upright optical microscope and a 63× Plan-Apochromat oil immersion objective (numerical aperture 1.4). GFP was excited at 475-nm with an Omega Set XF 100 filter by Attoarc HBO 100 epi-fluorescence lamp. The same acquisition parameters were used for all samples. AxioVision Release 4.6 software was used for image acquisition. Images were then prepared for publication using Adobe Photoshop™.

### Western analysis

The methods for western blot analysis have been described previously [26]. Rabbit anti-GFP antibody (SC-8334, Santa Cruz Technology, Santa Cruz, CA) was used to detect GFP-tagged proteins.

### Shmooing and mating

The methods for analyzing shmoo formation in response to 3×10^−6^ M α-pheromone, the synthetic 13-mer, were previously described in detail [13],[25]. The methods for testing mating with opaque α/α cells of strain WO-1 have also been described [9].

### Adhesion and biofilm formation

The methods for analyzing α-pheromone-induced adhesion to a plastic surface were previously described in detail [20],[24]. Adhesion was assessed after 16 hr on the surface of a plastic Costar twelve-well cluster plate (Corning Life Sciences, Lowell, MA). The analysis of white cell biofilm enhancement by minority opaque cells (5% opaque **a**/**a** P37005 cells and 5% opaque α/α WO-1 cells) was previously described [20]. Biofilm thickness was analyzed by laser scanning confocal microscopy of calcofluor-stained biofilms after 48 hr of incubation on a silicone elastomer surface. The intensity of calcofluor staining through the depth of a biofilm was represented as a graph in which the mean pixel intensity (y-axis) was plotted as a function of depth (x-axis). The mean grayscale value (0–256) of all the pixels (512×512) in each X-Y optical section was calculated. To visualize the extrapolymeric substance (EPS), also referred to as “matrix”, between cells in a biofilm, excitation of calcofluor was increased. Since the EPS was much dimmer than the cells in a biofilm, the laser power at 780 nm was increased to the point at which the cell pixels became saturated. Adobe Photoshop™ was then used to remove the saturated pixels (the cells), leaving behind an image of the EPS. Grayscale images were pseudocolored using Confocal Assistant software LUT (supplemental Figure S1).

### Generating PBR1-misexpression strains

The plasmid pNIM1 [42], harboring a GFP gene and the tetracycline-regulated promoter, was employed in this study. The pNIM1 plasmid was also a generous gift from Joachim Morschhäuser. The ORF of the *PBR1* gene, amplified by PCR with primers listed in supplemental Table S2, was digested with SalI and subcloned into the plasmid pNIM1 that had been digested with SalI and dephosphorylated, to derive pTet-PBR1. The correct orientation of the *PBR1* ORF was confirmed by sequencing. The GFP gene was fused in-frame to the C-terminus of *PBR1* ORF. The plasmid pTet-PBR1 was then digested with ApaI plus SacII, and transformed into either wild-type or mutant strains. The transformants were verified by PCR and Southern analysis. Activation of the *PBR1* transcription by doxycycline was demonstrated by northern analysis. We generated in this way the derivative strains P37005-tet*PBR1*, *EAP1_WPREΔ_/eap1*-tet*PBR1*, *PGA10_WPREΔ_*/*pga10*-tet*PBR1*, *CSH1_WPREΔ_/csh1*-tet*PBR1*, *PBR1_WPREΔ_/pbr1*-tet*PBR1* and *cek1cek2-tetPBR1*.

### Measurements of secreted (1, 3)-β-glucan concentration from biofilms

Equal numbers of cells (5×10^7^) of mutant or parental strains were cast without (-) and with (+) 1% opaque cells onto silicone elastomer squares in RPMI medium, as described previously [20],[25],[26]. After 48 hr, supernatants from biofilm culture were collected by pipetting off the supernatant without disturbing the culture, centrifuging at 4,000 rpm for 5 min, and removing the supernatant. Glucan concentration in the supernatants was then measured using Glucatell (1,3)-β-Glucan Detection Reagent Kit (Associates of Cape Cord, Falmouth, MA) [41]. Optical density (OD) values were determined at 540 nm in a microplate reader (MDS Analytical Technologies, Sunnyvale, CA) and the glucan concentration assessed by an end-point assay according to the manufacturer's protocol. Four biofilms were employed for each condition and strain. The means and standard deviation of glucan concentration are presented in a bar chart.

### Accession numbers

Detailed information for the genes from this study can be found at the *Candida* Genome Database http://www.candidagenome.org. The gene names and ORF numbers are listed here: *EAP1* (orf19.1401), *PGA10* (orf19.5674), *CSH1* (orf19.4477), *PBR1* (orf19.6274), RBT5 (orf19.5636), *LSP1* (orf19.3149), *PHR1* (orf19.3829), *PHR2* (orf19.6081), *SUN41* (orf19.3642), *WH11* (orf19.3548.1), orf19.2077, *CIT1* (orf19.4393), *STE2* (orf19.696), *CEK1* (orf19.2886), *CEK2* (orf19.460), *SST2* (orf19.4222), *RBT1* (orf19.1327), *MFA1* (orf19.2164.1), *FUS1* (orf19.1156), *CPH1* (orf19.4433), *ECE1* (orf19.3374), *KAR4* (orf19.3736), *RAM1* (orf19.5046).

## Supporting Information

Figure S1 The matrix of control cell biofilms was far more pronounced than that of the four mutants. For pseudocolor, red represents cells, yellow-green represents matrix and blue represents open space. See Materials and Methods for protocol.
(3.11 MB TIF)

Figure S2 Deletion of the lcWPRE from the promoter of the strains *CSH1_WPREΔ_/csh1* and *PBR1_WPREΔ_/pbr1* does not remove the low level of adhesion induced by α-pheromone. Quantitation of cells adherent to the well bottoms in the absence (−) and presence (+) of α-pheromone (α-ph) was performed according to the protocol described in Materials and Methods. The means and standard deviations (error bars) of three independent samples are presented in a bar chart.
(0.10 MB TIF)

Table S1 Genes screened for differential expression in *C. albicans* white cells in response to pheromone.
(0.06 MB DOC)

Table S2 Oligonucleotides used for generating mutants in this study.
(0.09 MB DOC)

Table S3 Genes analyzed by northern blot hybridization in this study and oligonucleotides.
(0.19 MB DOC)

Table S4 *C. albicans* strains used.
(0.06 MB DOC)

Table S5 The white-specific pheromone response elements (WPRE) found in genes up-regulated by α-pheromone exclusively in white cells and in genes up-regulated by pheromone in both white and opaque cells.
(0.06 MB DOC)

Table S6 The opaque-specific pheromone response elements (OPRE) found in genes up-regulated by α-pheromone exclusively in opaque cells and in genes up-regulated by pheromone both in white and opaque cells.
(0.06 MB DOC)

Table S7 The significance of the differences in biofilm thickness between complemented controls and deletion mutants, described in Figure 6.
(0.04 MB DOC)
