# Supplementary figures and images for: Genes Selectively Up-Regulated by Pheromone in White Cells Are Involved in Biofilm Formation in Candida albicans
Source: PLoS Pathog. 2009 Oct 2;5(10):e1000601. doi: 10.1371/journal.ppat.1000601 (PMC2745568; doi:10.1371/journal.ppat.1000601)

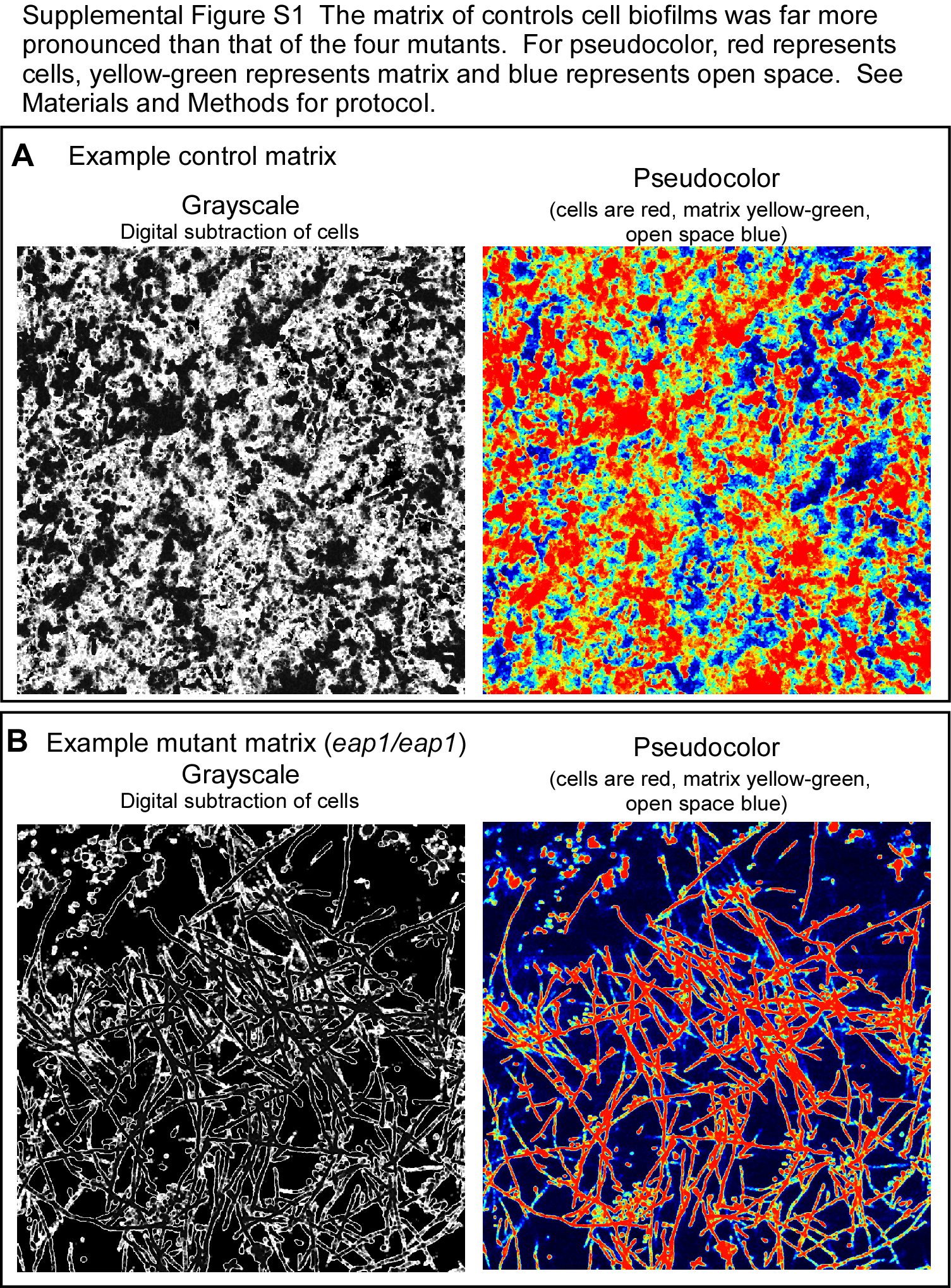

Supplement: Figure S1 — The matrix of control cell biofilms was far more pronounced than that of the four mutants. For pseudocolor, red represents cells, yellow-green represents matrix and blue represents open space. See Materials and Methods for protocol. (3.11 MB TIF) [file ppat.1000601.s001.tif]

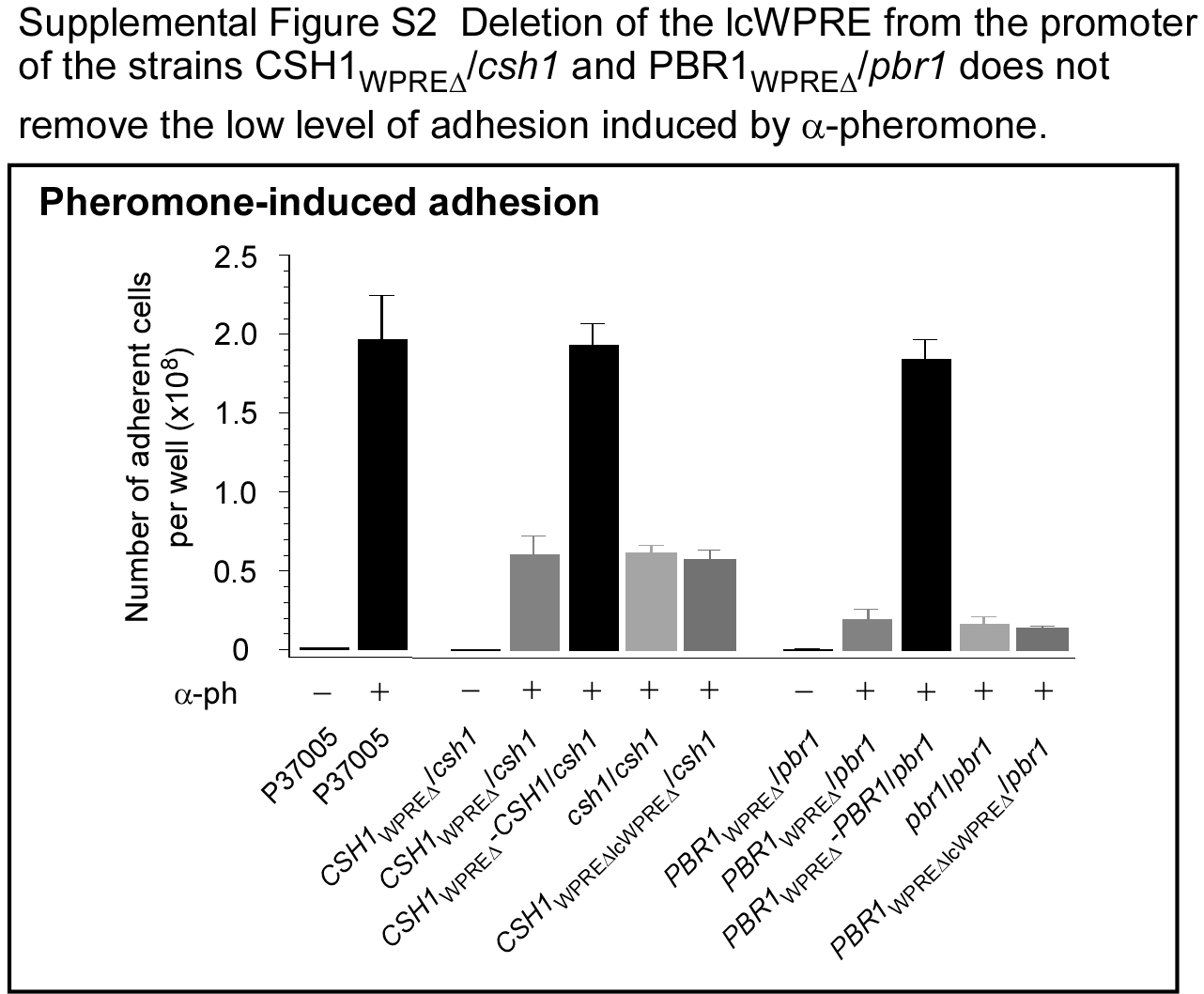

Supplement: Figure S2 — Deletion of the lcWPRE from the promoter of the strains CSH1WPREΔ/csh1 and PBR1WPREΔ/pbr1 does not remove the low level of adhesion induced by α-pheromone. Quantitation of cells adherent to the well bottoms in the absence (−) and presence (+) of α-pheromone (α-ph) was performed according to the protocol described in Materials and Methods. The means and standard deviations (error bars) of three independent samples are presented in a bar chart. (0.10 MB TIF) [file ppat.1000601.s002.tif]
