# Supplementary material for: Genes Selectively Up-Regulated by Pheromone in White Cells Are Involved in Biofilm Formation in Candida albicans
Source: PLoS Pathog. 2009 Oct 2;5(10):e1000601. doi: 10.1371/journal.ppat.1000601 (PMC2745568; doi:10.1371/journal.ppat.1000601)
Supplement: Table S1 — Genes screened for differential expression in C. albicans white cells in response to pheromone. (0.06 MB DOC) [file ppat.1000601.s003.doc]

# Supporting information

| **Supplemental Table S1. Genes screened for differential expression in *C. albicans* white cells in response to pheromone** | | | | | | | |  |
| --- | --- | --- | --- | --- | --- | --- | --- | --- |
|  |
|  | | | | | | | |  |
| **A. Genes strongly induced by pheromone** | | | | | | | |  |
| Adhesion: | *EAP1* | *PGA10* | *RBT5* |  |  | |  |  |
| Cell wall biogenesis | *PHR1* | *PHR2* |  |  |  | |  |
| Biofilm formation: | *CSH1* | *LSP1* | *CIT1* | *SUN41* |  | |  |
| Other: | *PBR1* | *WH11* | *19.2077* |  |  | |  |  |
|  | | | | | | | |  |
|  | | | | | | | |  |
| **B. Genes slightly induced by pheromone** | | | |  | | | |  |
| Adhesion: | *INT1* |  |  |  |  |  | |  |
| Cell wall biogenesis: | *UTR2* | *PDE2* | *SSA2* | *OCH1* | *GPI8* | |  |  |
| Filamentation: | *GPA2* | *FGR23* | *RBT4* |  |  |  | |  |
|  | | | | | | | |  |
|  | | | | | | | |  |
| **C. Genes not induced by pheromone** | | | |  | | | |  |
| Adhesion: | *ALS1* | *ALS2* | *ALS3* | *ALS4* | *ALS5* | | *ALS6* |  |
|  | *ALS7* | *ALS9* | *HYR1* | *IFF4* | *CSA1* | | *ECM33* |
|  | *HSP12* | *ECM331* | *AAF1* | *SAP1* | *ECE1* | | *BGL2* |
|  | *PGA59* | *MSB1* | *CSE4* |  |  | |  |  |
| Cell wall biogenesis: | *CHK1* | *KRE1* | *SSK1* | *SMI1B* | *CRH1* | | *IFF11* |  |
|  | *GPI1* | *GPI13* | *CHS1* | *EXG1* | *UAP1* | | *RAM1* |  |
| Biofilm formation: | *PMT1* | *ACE2* | *PMT5* | *MDR1* | *ENO1* | | *ADH1* |  |
|  | *RIX7* | *MIG1* | *CDR1* | *CDR3* | *YWP1* | | *VPS1* |
|  | *NUP85* | *KEM1* | *SUV3* | *SNF1* |  | |  |
| Filamentation | *RNH1* | *CSC25* | *NAG2* | *SHE3* | *RBF1* | | *VAC1* |
|  | *RFG1* | *DDR48* | *IRS4* | *IHD1* | *REG1* | | *MNN2* |
|  | *SWI1* | *NOT4* | *ADR1* | *CRK1* | *RAS1* | | *RAS2* |
|  | *PTC1* | *BIG1* | *PLD1* | *HSL1* | *NOT3* | | *YVH1* |
|  | *SPT6* | *FIG1* | *RAX2* | *UPC2* | *CPH1* | | *KEL1* |
|  | *CDC5* | *GAL10* | *HXK1* |  |  | |  |
