# Supplementary material for: Genes Selectively Up-Regulated by Pheromone in White Cells Are Involved in Biofilm Formation in Candida albicans
Source: PLoS Pathog. 2009 Oct 2;5(10):e1000601. doi: 10.1371/journal.ppat.1000601 (PMC2745568; doi:10.1371/journal.ppat.1000601)
Supplement: Table S2 — Oligonucleotides used for generating mutants in this study. (0.09 MB DOC) [file ppat.1000601.s004.doc]

| **Supporting information** | |  |
| --- | --- | --- |
|  |  |  |
| **Supplemental Table S2. Oligonucleotides used for generating mutants in this study** | | |
|  |  |  |
| **Primer** | **Gene/Purpose** | **Sequence** |
| EAP1f1 | *EAP1* heterozygote | 5'-TACCTTTTAGTACTCTGT-3' |
| EAP1r1 | *EAP1* heterozygote | 5'-TCCCCCGGGCAACTGAAGAGATCTTGA-3' |
| EAP1f2 | *EAP1* heterozygote | 5'-TCCCCCGGGTGTACCATATGGTGGTGA-3' |
| EAP1r2 | *EAP1* heterozygote | 5'-TCCTGTTGTTGATGTTCA-3' |
| PGA10f1 | *PGA10* heterozygote | 5'-TTCATTATGGACCCATTT-3' |
| PGA10r1 | *PGA10* heterozygote | 5'-TCCCCCGGGTTGCGAGAATAAGTTTGT-3' |
| PGA10f2 | *PGA10* heterozygote | 5'-TCCCCCGGGGTTTTTCATTAAATGATGAGA-3' |
| PGA10r2 | *PGA10* heterozygote | 5'-TCTAAGAAGAAGCCAGAT-3' |
| CSH1f1 | *CSH1* heterozygote | 5'-AAGTTGATGCTTTATCAG-3' |
| CSH1r1 | *CSH1* heterozygote | 5'-TCGCCCGGGTCGGAACAATTACTGTAT-3' |
| CSH1f2 | *CSH1* heterozygote | 5'-TCGCCCGGGTAACGAAATAATTTGTCA-3' |
| CSH1r2 | *CSH1* heterozygote | 5'-TGACATATTTTGGTTAGA-3' |
| PBR1f1 | *PBR1* heterozygote | 5'-TTCCATCAACCAGTTGCT-3' |
| PBR1r1 | *PBR1* heterozygote | 5'-TCCCCCGGGTTGTTGAGGTTTCAGTTT-3' |
| PBR1f2 | *PBR1* heterozygote | 5'-TCCCCCGGGTGTTGCCTATCCAAATTG-3' |
| PBR1r2 | *PBR1* heterozygote | 5'-TGATTATGTAATAAACTCCA-3' |
| EAP1pf1 | WPRE deletion | 5'-AGCCAAGTTTATACACAT-3' |
| EAP1pr1 | WPRE deletion | 5'-TCCCCCGGGCTATCATGCAACAGTGAT-3' |
| EAP1pf2 | WPRE deletion | 5'-TCCCCCGGGGTCAATTAATTAGCTAGAT-3' |
| EAP1pdelf | WPRE deletion | 5'-CAATGAGGTGAACTTAATAAAAGGGTTCTA-3' |
| EAP1pdelr | WPRE deletion | 5'-TTATTAAGTTCACCTCATTGTATTAATTC-3' |
| EAP1pr2 | WPRE deletion | 5'-TCCTGTTGTTGATGTTCA-3' |
| PGA10pf1 | WPRE deletion | 5'-CCAAATCGATCTAGAGGT-3' |
| PGA10pr1 | WPRE deletion | 5'-TCCCCCGGGTTAACATCAGTCTCCAGT-3' |
| PGA10pf2 | WPRE deletion | 5'-TCCCCCGGGACAGAAATTGGAGATGTC-3' |
| PGA10pdelf | WPRE deletion | 5'-GATAGAGAATATGGCTGTTGTTCAATTC-3' |
| PGA10pdelr | WPRE deletion | 5'-CAACAGCCATATTCTCTATCCGAGAATC-3' |
| PGA10pr2 | WPRE deletion | 5'-GTTTGTACCGTCTGAAGT-3' |
| CSH1pf1 | WPRE deletion | 5'-TTTTGCTCTTGTAGACAC-3' |
| CSH1pr1 | WPRE deletion | 5'-TCCCCCGGGACCTCAACAGTATAAACA-3' |
| CSH1pf2 | WPRE deletion | 5'-TCCCCCGGGTCGATGGTTCAGTATATT-3' |
| CSH1pdelf | WPRE deletion | 5'-TATCGATTGAAGTTAAAGTTAAGTTGTAGTT-3' |
| CSH1pdelr | WPRE deletion | 5'-AACTTTAACTTCAATCGATAAATCAAGAAT-3' |
| CSH1pr2 | WPRE deletion | 5'-TAGACTTCTCAACAACCC-3' |
| PBR1pf1 | WPRE deletion | 5'-AGGAAGATGACAATTCAT-3' |
| PBR1pr1 | WPRE deletion | 5'-TCCCCCGGGAGGAGAAAACATACACAAA-3' |
| PBR1pf2 | WPRE deletion | 5'-TCCCCCGGGACTTATTGATACACCAAT-3' |
| PBR1pdelf | WPRE deletion | 5'-TGGTTATACCAGAGTTACCAATTGGTTA-3' |
| PBR1pdelr | WPRE deletion | 5'-TGGTAACTCTGGTATAACCAACTAATAC-3' |
| PBR1pr2 | WPRE deletion | 5'-TAGTAGTTGTAATTAATGAATTAAT-3' |
| EAP1wQ1f | WPRE complementation | 5'-TTCCATTTCTATAGGCTC-3' |
| EAP1wQ1r | WPRE complementation | 5'-TCCGGATCCCATAAAGTAGACTAATGC-3' |
| EAP1wQ2f | WPRE complementation | 5'-TCCGGATCCTGTACCATATGGTGGTGA-3' |
| EAP1wQ2r | WPRE complementation | 5'-TCCTGTTGTTGATGTTCA-3' |
| PGA10wQ1f | WPRE complementation | 5'-TGATGAACGTGGTATGAA-3' |
| PGA10wQ1r | WPRE complementation | 5'-TCCGGATCCGATTAAGGCAGCAAATGC-3' |
| PGA10wQ2f | WPRE complementation | 5'-TCCGGATCCCTACTGTTAATAGGTGAT-3' |
| PGA10wQ2r | WPRE complementation | 5'-AGAACCGTCAGCATATAA-3' |
| CSH1wQ1f | WPRE complementation | 5'-ATCTCTGTGCAATGTGAA-3' |
| CSH1wQ1r | WPRE complementation | 5'-TCCAGATCTAGCAGCAACTCTTGCCAA-3' |
| CSH1wQ2f | WPRE complementation | 5'-TCCAGATCTCTAAGTGATTCATAAGGA-3' |
| CSH1wQ2r | WPRE complementation | 5'-CACGGTTAGAATTCATTT-3' |
| PBR1wQ1f | WPRE complementation | 5'-CCATCATTACATGGTGAT-3' |
| PBR1wQ1r | WPRE complementation | 5'-TCCGGATCCCAAGACAGCCCAATTGAGA-3' |
| PBR1wQ2f | WPRE complementation | 5'-TCCGGATCCTGTTGCCTATCCAAATTG-3' |
| PBR1wQ2r | WPRE complementation | 5'-TGATTATGTAATAAACTCCA-3' |
| SATBgF1 | *GFP-SAT1* PCR | 5'-TCAAGATCTTCCATCATAAAATGTCGA-3' |
| GFBhF1 | *GFP-SAT1* PCR | 5'-TCAGGATCCATGTCTAAAGGTGAAGAA-3' |
| EAP1f3 | *EAP1* homozygote | 5'-AGAGAAGATAGAACCCTT-3' |
| EAP1r3 | *EAP1* homozygote | 5'-TCCCCCGGGAGCTGGAGTACTTTCAGT-3' |
| EAP1f4 | *EAP1* homozygote | 5'-TCCCCCGGGTTATCCCAGGCACTGAAA-3' |
| EAP1r4 | *EAP1* homozygote | 5'-AGGTGATGGTGATAATCA-3' |
| PGA10f3 | *PGA10* homozygote | 5'-TCGCTTAAAATCCGAACA-3' |
| PGA10r3 | *PGA10* homozygote | 5'-TCCCCCGGGCCAATAGCACCACCAAAT-3' |
| PGA10f4 | *PGA10* homozygote | 5'-TCCCCCGGGCATGAAAGCAAAGTAGCT-3' |
| PGA10r4 | *PGA10* homozygote | 5'-GAAAACATTGGATAACAC-3' |
| CSH1f3 | *CSH1* homozygote | 5'-TGTTCCGATAACCACCACT-3' |
| CSH1r3 | *CSH1* homozygote | 5'-TCGCCCGGGTCAAGTGAACTGTCTTCT-3' |
| CSH1f4 | *CSH1* homozygote | 5'-TCGCCCGGGAGAGACGCAGATAAGATT-3' |
| CSH1r4 | *CSH1* homozygote | 5'-CAGTTTCAACAAATGGAAT-3' |
| PBR1f3 | *PBR1* homozygote | 5'-AACACACAGCTTTATAGT-3' |
| PBR1r3 | *PBR1* homozygote | 5'-TCCCCCGGGCTTACCATTGACTTCTTC-3' |
| PBR1f4 | *PBR1* homozygote | 5'-TCCCCCGGGAACCAGCTTATTCTGCTA-3' |
| PBR1r4 | *PBR1* homozygote | 5'-CAAATCAACGAGAGATCA-3' |
| TetPBR1f | *PBR1* overexpression | 5'-TCCGTCGACAAAGATGTACAAATTCACTGTT-3' |
| TetPBR1r | *PBR1* overexpression | 5'-TCCGTCGACAACAAGACAGCCCAATTGAG-3' |
