# Supplementary material for: Genes Selectively Up-Regulated by Pheromone in White Cells Are Involved in Biofilm Formation in Candida albicans
Source: PLoS Pathog. 2009 Oct 2;5(10):e1000601. doi: 10.1371/journal.ppat.1000601 (PMC2745568; doi:10.1371/journal.ppat.1000601)
Supplement: Table S3 — Genes analyzed by northern blot hybridization in this study and oligonucleotides. (0.19 MB DOC) [file ppat.1000601.s005.doc]

| **Supporting information** | |  |
| --- | --- | --- |
|  |  |  |
| **Supplemental Table S3. Genes analyzed by northern blot hybridization in this study and oligonucleotides** | | |
|  |  |  |
| **Gene** | **Primer** | **Sequence** |
| *EAP1* | EAP1f | 5'-TAGCACTGATTGTACCAC-3' |
| *EAP1* | EAP1r | 5'-AGCTGGTGTAGATTCAGT-3' |
| *PGA10* | PGA10f | 5’-TGACTGTGTTGCTAAAAA-3’ |
| *PGA10* | PGA10r | 5’-TGCTGCGGAGGACTTTTC-3’ |
| *CSH1* | CSH1f | 5'-TCGACTCTGAAAAAACTA-3' |
| *CSH1* | CSH1r | 5'-CATGCCAATGAAACTTGC-3' |
| *PBR1* | PBR1f | 5’-AATGTGACTTTATACATT-3’ |
| *PBR1* | PBR1r | 5’-CAGCATATAAGTAATCAT-3’ |
| *RBT5* | RBT5f | 5'-TGATGCCGCTGCTGAAAC-3' |
| *RBT5* | RBT5r | 5'-ACAGCGGCAATGACACCA-3' |
| *PHR1* | PHR1f | 5'-TCATGAAGAGTGTATGAA-3' |
| *PHR1* | PHR1r | 5'-TACATCCATATTCGGAGA-3' |
| *PHR2* | PHR2f | 5'-TGAATCCATCAACAGAGA-3' |
| *PHR2* | PHR2r | 5'-ATACCACCAGACCAAACT-3' |
| *LSP1* | LSP1f | 5'-TGGTGAATTGGAAGATCA-3' |
| *LSP1* | LSP1r | 5'-CTTTACCATAACCAGCAA-3' |
| *CIT1* | CIT1f | 5'-AACTGTCATTGGTGAAGT-3' |
| *CIT1* | CIT1r | 5'-ATTCCTTGTTGTCACCAA-3' |
| *SUN41* | SUN41f | 5’-GCTTGCCAAAGTGGTATG-3’ |
| *SUN41* | SUN41r | 5’-ACCTCTCCAAGTGTAATA-3’ |
| *WH11* | WH11f | 5'-ATGTCCGACTTAGGTAGA-3' |
| *WH11* | WH11r | 5'-TTATTTGGAGTCACCAAA-3' |
| *19.2077* | 19.2077f | 5'-TTAAAAGGAGCCAAGAGT-3' |
| *19.2077* | 19.2077r | 5'-TCAACACCTTTGTCGTCA-3' |
| *INT1* | INT1f | 5'-CAAACCGAGTTTAGATCC-3' |
| *INT1* | INT1r | 5'-TGACACTTTAGGAGTTGC-3' |
| *UTR2* | UTR2f | 5'-AGTTAAAGAAATTGAATTA-3' |
| *UTR2* | UTR2r | 5'-TCTTTAATGTGAGCATAA-3' |
| *PDE2* | PDE2f | 5'-TACTACTACTACTAATAC-3' |
| *PDE2* | PDE2r | 5'-AATAAACCAAATCGTCAT-3' |
| *SSA2* | SSA2f | 5’-GTTCAGATCTACTTTGGA-3’ |
| *SSA2* | SSA2r | 5’-TTTGGTCATGATACCACC-3’ |
| *OCH1* | OCH1f | 5’-GATTAAAACCAGTTGATG-3’ |
| *OCH1* | OCH1r | 5’-TCAGTGAAAATCCCAGGA-3’ |
| *GPI8* | GPI8f | 5’-AGGTATAACGAAATATTT-3’ |
| *GPI8* | GPI8r | 5’-CTTCACTAACATTACGCT-3’ |
| *GPA2* | GPA2f | 5'-AATGAAATTTTAGATTAT-3' |
| *GPA2* | GPA2r | 5'-GACCACCAACATCAAATA-3' |
| *FGR23* | FGR23f | 5’-TACTCATTGTCCTGAATG-3’ |
| *FGR23* | FGR23r | 5’-AGTTGGACATGTAGTTAT-3’ |
| *RBT4* | RBT4f | 5'-TCTACTAAAGACGCTTCT-3' |
| *RBT4* | RBT4r | 5'-GTACCAAGCTTGAAGAGC-3' |
| *ALS1* | ALS1f | 5'-CATGTCAAGTTCAAAGAT-3' |
| *ALS1* | ALS1r | 5'-CTAGTTACGATTGAGGAT-3' |
| *ALS2* | ALS2f | 5'-CCGGGTGGTACTGACTCA-3' |
| *ALS2* | ALS2r | 5'-ATTCAGTAGTGGTCACAG-3' |
| *ALS3* | ALS3f | 5'-TATGACACCATGTCAAGT-3' |
| *ALS3* | ALS3r | 5'-AGCAGTAGTAAAAGTAGA-3' |
| *ALS4* | ALS4f | 5'-CACCAATAGTGTCATTAT-3' |
| *ALS4* | ALS4r | 5'-ATAGAAGTTTGACAACCA-3' |
| *ALS5* | ALS5f | 5'-TACAACTACAACCCAGTT-3' |
| *ALS5* | ALS5r | 5'-TTCTCTAACAATGACTGA-3' |
| *ALS6* | ALS6f | 5'-TCAAGTATGAGTTGTGTA-3' |
| *ALS6* | ALS6r | 5'-AATCCGAGCACACCGGAT-3' |
| *ALS7* | ALS7f | 5'-TCACGTAGAGTCCAGTAGT-3' |
| *ALS7* | ALS7r | 5'-TGACGTAGTGTACAAATC-3' |
| *ALS9* | ALS9f | 5'-ACCAAGTGTTTCCAGTTT-3' |
| *ALS9* | ALS9r | 5'-AATGGAAGTGACCGCACT-3' |
| *HYR1* | HYR1f | 5'-TCTCAACCTCAGTGCTGC-3' |
| *HYR1* | HYR1r | 5'-AACAAGACCCGAAGAAG-3' |
| *IFF4* | IFF4f | 5'-TCTTCTACAGCCGGAAGT-3' |
| *IFF4* | IFF4r | 5'-TTCACTGGAGACAAATGA-3' |
| *CSA1* | CSA1f | 5'-GCAGAAGCTGCTCATAAG-3' |
| *CSA1* | CSA1r | 5'-AAACACGAAACTAGCGAC-3' |
| *ECM33* | ECM33f | 5'-ATGCAAATTAAGTCATTTCT-3' |
| *ECM33* | ECM33r | 5'-GATTGAAATTTAATGAGAC-3' |
| *HSP12* | HSP12f | 5'-ACAAACACCATAAATCCC-3' |
| *HSP12* | HSP12r | 5'-CATATTCTTGAGCTGTTT-3' |
| *ECM331* | ECM331f | 5’-CGGTAATTTAACTATTCA-3’ |
| *ECM331* | ECM331r | 5’-TTCCATAGTCAAATTATC-3’ |
| *AAF1* | AAF1f | 5’-TGATGTAACGGTCAATTT-3’ |
| *AAF1* | AAF1r | 5’-ATTTTTATTTGTCAGTTT-3’ |
| *SAP1* | SAP1f | 5’-AGGAGTTATTGCCAAGAA-3’ |
| *SAP1* | SAP1r | 5’-GAAAGTATGACCTTGACC-3’ |
| *ECE1* | ECE1f | 5’-TAATGCCGTCGTCAGATT-3’ |
| *ECE1* | ECE1r | 5’-ATTGCTAAGTGCTACTGA-3’ |
| *BGL2* | BGL2f | 5’-CAAGTGTCCCAGTTGGTA-3’ |
| *BGL2* | BGL2r | 5’-CAGATACCTTTTTGCCAT-3’ |
| *PGA59* | PGA59f | 5'-ATGCAATTCTCATCCGCT-3' |
| *PGA59* | PGA59r | 5'-AACAAACCGGCAGCAACG-3' |
| *MSB1* | MSB1f | 5’-CTGTTTCTTAATCACAGA-3’ |
| *MSB1* | MSB1r | 5’-TAAACTCAGAATGTCATT-3’ |
| *CSE4* | CSE4f | 5’-ATCATCGCTACCACGAAG-3’ |
| *CSE4* | CSE4r | 5’-TGAATCGCACATAAATTT-3’ |
| *CHK1* | CHK1f | 5'-TACCAGACCAATATGAAC-3' |
| *CHK1* | CHK1r | 5'-TTAGTGCAACAGTTGTAT-3' |
| *KRE1* | KRE1f | 5'-TGAACGTTAACTCAGTCA-3' |
| *KRE1* | KRE1r | 5'-TGAATGAAATGAATGCAA-3' |
| *SSK1* | SSK1f | 5'-ACTACTAGAAAGAACAGA-3' |
| *SSK1* | SSK1r | 5'-TAAACCTACACGAGTATT-3' |
| *SMI1B* | SMI1Bf | 5'-ACCAAATTGTGTCATTCA-3' |
| *SMI1B* | SMI1Br | 5'-ACATTGAAGTTGCGGTAT-3' |
| *CRH1* | CRH1f | 5'-ATGATTTGGATGAAATTG-3' |
| *CRH1* | CRH1r | 5'-ATACTTGTGTCCTTCATA-3' |
| *IFF11* | IFF11f | 5’-GTCTATTACTATTGCTGG-3’ |
| *IFF11* | IFF11r | 5’-ACTAGTAGTTTTTGATTG-3’ |
| *GPI1* | GPI1f | 5’-TTACCTCAATTATTAAAT-3’ |
| *GPI1* | GPI1r | 5’-ATTAAATTAATCCCATCA-3’ |
| *GPI13* | GPI13f | 5’-TACGAGTACTGACAATGT-3’ |
| *GPI13* | GPI13r | 5’-ACATATGAATTAGATGCA-3’ |
| *CHS1* | CHS1f | 5’-CAGGGGCTGCTGGTGAAA-3’ |
| *CHS1* | CHS1r | 5’-GCCAAGTACATATTTGCT-3’ |
| *EXG1* | EXG1f | 5’-GTTGTTATTGGTATTGAA-3’ |
| *EXG1* | EXG1r | 5’-ACGTTCCAATGGGATTCC-3’ |
| *UAP1* | UAP1f | 5’-ATTCTAAGGGCATCAAAC-3’ |
| *UAP1* | UAP1r | 5’-TTTAAAAATTCCACTGAA-3’ |
| *RAM1* | RAM1f | 5’-TAGATAATTTAAGAGAAT-3’ |
| *RAM1* | RAM1r | 5’-GCAAGGGCACAATAAGTA-3’ |
| *PMT1* | PMT1f | 5'-GGCAAAGAAACCGTCACA-3' |
| *PMT1* | PMT1r | 5'-ATCAGGAAATTTATCACCA-3' |
| *ACE2* | ACE2f | 5'-TCCATTAACGGTGTCAACA-3' |
| *ACE2* | ACE2r | 5'-TTGGCAACAATGTACTCGT-3' |
| *PMT5* | PMT5f | 5’-TGATGACTGATCAAAGAG-3’ |
| *PMT5* | PMT5r | 5’-AGTTTCTGTTCGTGACTC-3’ |
| *MDR1* | MDR1f | 5'-TAATGATAATGATGTTGAT-3' |
| *MDR1* | MDR1r | 5'-TTTAACCACATCAGCAAC-3' |
| *ENO1* | ENO1f | 5’-TGGTAACGTCGGTGACGA-3’ |
| *ENO1* | ENO1r | 5’-GTGGACCCAAGCATCCCA-3’ |
| *ADH1* | ADH1f | 5'-ATGCAAGCAAGCTTATTC-3' |
| *ADH1* | ADH1r | 5'-TATCAAAGACAACGGCTT-3' |
| *RIX7* | RIX7f | 5’-CAGGTATAAGTGCTATTA-3’ |
| *RIX7* | RIX7r | 5’-GGAATAGTTGCGAATCCC-3’ |
| *MIG1* | MIG1f | 5’-CCGATGGTAATAAATTAT-3’ |
| *MIG1* | MIG1r | 5’-TTAGTATTAGATGTTGTT-3’ |
| *CDR1* | CDR1f | 5’-GCTGGTGCTTATCAATAT-3’ |
| *CDR1* | CDR1r | 5’-CTGGGAAATCAACACTTC-3’ |
| *CDR3* | CDR3f | 5’-GTATTGATGTGGTTAATC-3’ |
| *CDR3* | CDR3r | 5’-TCCTGACATTTTTGAACT-3’ |
| *YWP1* | YWP1f | 5’-ACTGTCCATTAAGTTCTT-3’ |
| *YWP1* | YWP1r | 5’-TTATAAGTAACATAATGA-3’ |
| *VPS1* | VPS1f | 5’-TCCTGCATCAATTGCATT-3’ |
| *VPS1* | VPS1r | 5’-ACATCTGATAGAAGGTTC-3’ |
| *NUP85* | NUP85f | 5’-CCGAAAAGCCGCCTTCGG-3’ |
| *NUP85* | NUP85r | 5’-GGTACCAATTCATGAATA-3’ |
| *KEM1* | KEM1f | 5’-TCAACCATTCCATTTACT-3’ |
| *KEM1* | KEM1r | 5’-TAGAAACTTCGGAACGGA-3’ |
| *SUV3* | SUV3f | 5’-AGTGGAAAAACTGAATAC-3’ |
| *SUV3* | SUV3r | 5’-AATTTTTCTACAGTAGAA-3’ |
| *SNF1* | SNF1f | 5’-TATGCCAGATTATTTGTT-3’ |
| *SNF1* | SNF1r | 5’-TGGACTTGGTGGTGGAGA-3’ |
| *RNH1* | RNH1f | 5'-ATGCCATATTACGCAGTT-3' |
| *RNH1* | RNH1r | 5'-TCAGTATCTTCCTGTAGT-3' |
| *CSC25* | CSC25f | 5’-GGTTAACTTATAAGTCGC-3’ |
| *CSC25* | CSC25r | 5’-ATAGAATAAAGGATCTTC-3’ |
| *NAG2* | NAG2f | 5’-TCGGATTATGACACAGCC-3’ |
| *NAG2* | NAG2r | 5’-ACCGATCGCCAGTCTTTA-3’ |
| *SHE3* | SHE3f | 5’-CTTGTCGATTTTGAATGA-3’ |
| *SHE3* | SHE3r | 5’-GAAACTTTTTCAATCATA-3’ |
| *RBF1* | RBF1f | 5’-TGAAAAGGATCAGAAAAG-3’ |
| *RBF1* | RBF1r | 5’-TTGCTGTTGTGCTTGGCT-3’ |
| *VAC1* | VAC1f | 5’-AGTTGAATTACACACACG-3’ |
| *VAC1* | VAC1r | 5’-CGTTCATTGTCTCTTGTG-3’ |
| *RFG1* | RFG1f | 5’-CTTCCTAATAATAAATCT-3’ |
| *RFG1* | RFG1r | 5’-AGTTTGTGGGTTGCTAGT-3’ |
| *DDR48* | DDR48f | 5’-AGAGAAGTAACGATTCAT-3’ |
| *DDR48* | DDR48r | 5’-TGTTGGAAGAGCCATAGG-3’ |
| *IRS4* | IRS4f | 5’-CAGTTTCTAGTCGTCTTC-3’ |
| *IRS4* | IRS4r | 5’-GATCCATATCACGATAGT-3’ |
| *IHD1* | IHD1f | 5’-CAAATGAAACTGGTTCTG-3’ |
| *IHD1* | IHD1r | 5’-CTGGTTCCATTAGTAAAT-3’ |
| *REG1* | REG1f | 5’-AATAATTTTGGGCCAGCA-3’ |
| *REG1* | REG1r | 5’-ATAAGATGCATACACTGA-3’ |
| *MNN2* | MNN2f | 5’-TGTTTGAAAGTGATTTAT-3’ |
| *MNN2* | MNN2r | 5’-TTGTAGTATAAACAAAGC-3’ |
| *SWI1* | SWI1f | 5’-TCATGGAGTTATTAGACG-3’ |
| *SWI1* | SWI1r | 5’-TGAAACTCAAGCACATCT-3’ |
| *NOT4* | NOT4f | 5’-CGAAGCCCATAAGGAACA-3’ |
| *NOT4* | NOT4r | 5’-GCGTACATTTCTTATATC-3’ |
| *ADR1* | ADR1f | 5’-CATCCTTCTAAATTGAAT-3’ |
| *ADR1* | ADR1r | 5’-TCCGTAAAGAACAGACAA-3’ |
| *CRK1* | CRK1f | 5’-GATAAAGAACGATTTAAA-3’ |
| *CRK1* | CRK1r | 5’-GAATATCCCTCCGTCTTT-3’ |
| *RAS1* | RAS1f | 5’-TAGAAACTTCTGCTAAAC-3’ |
| *RAS1* | RAS1r | 5’-GGAAGATTTGCTTGACCA-3’ |
| *RAS2* | RAS2f | 5’-GCAAACGTAATGGGAGTT-3’ |
| *RAS2* | RAS2r | 5’-CGAGTTAGTGTTGATAAT-3’ |
| *PTC1* | PTC1f | 5’-TACTGCTGCAGTGGCTGT-3’ |
| *PTC1* | PTC1r | 5’-CATATATGTGTTCACCAAG-3’ |
| *BIG1* | BIG1f | 5’-GATGATCGAGTCAAAGAA-3’ |
| *BIG1* | BIG1r | 5’-TTCATCTTTCTTTTTATT-3’ |
| *PLD1* | PLD1f | 5’-CATGTGGGCAGACCGCAA-3’ |
| *PLD1* | PLD1r | 5’-TCGATGAAACAAGCTGTT-3’ |
| *HSL1* | HSL1f | 5’-GCACCATCTTCTGGTATG-3’ |
| *HSL1* | HSL1r | 5’-CATTAGTATCAGTTGTAC-3’ |
| *NOT3* | NOT3f | 5’-CAAACACTTCCTCATCAA-3’ |
| *NOT3* | NOT3r | 5’-GTCTTCAGTTCTACCAGT-3’ |
| *YVH1* | YVH1f | 5’-AATGAAATATTGAAGAGC-3’ |
| *YVH1* | YVH1r | 5’-TCTAATTCTTGTTTCATC-3’ |
| *SPT6* | SPT6f | 5’-GGGTCAAAGGCGAATGTT-3’ |
| *SPT6* | SPT6r | 5’-GTCAAATTCACTCTTTAC-3’ |
| *FIG1* | FIG1f | 5’-TTTAGCAATATATGTCAT-3’ |
| *FIG1r* | FIG1r | 5’-CATCAATATAAAACTAAA-3’ |
| *RAX2* | RAX2f | 5’-GTCTTAATAATACTTTTG-3’ |
| *RAX2* | RAX2r | 5’-AATTATTGAAATTACCAC-3’ |
| *UPC2* | UPC2f | 5’-TCAATATGCTTGACTTGA-3’ |
| *UPC2* | UPC2r | 5’-ATATCAAGGCACTGGCAA-3’ |
| *CPH1* | CPH1f | 5’-CAATTACGATTCATTTTT-3’ |
| *CPH1* | CPH1r | 5’-TGCTGAAATTGGCGGCAC-3’ |
| *KEL1* | KEL1f | 5’-CGACTTGCCTTCAACTAC-3’ |
| *KEL1* | KEL1r | 5’-GACCAATTCGTTGGTGAG-3’ |
| *CDC5* | CDC5f | 5’-CTCAACCAGGTGTGCTTT-3’ |
| *CDC5* | CDC5r | 5’-AGCTGGAGGGTTTGCAAG-3’ |
| *GAL10* | GAL10f | 5’-GGAAAAGTGTACAAATTA-3’ |
| *GAL10* | GAL10r | 5’-TGAGTTGGAAACATTGAA-3’ |
| *HXK1* | HXK1f | 5’-TTGGAAACAACCGACTAC-3’ |
| *HXK1* | HXK1r | 5’-CGTAGCATCAGCCAACAT-3’ |
| *STE2* | STE2f | 5'-GTGTTCAACATAAGAAGA-3' |
| *STE2* | STE2r | 5'-ATTATTAGCAGTTTGAGC-3' |
| *MFA1* | MFA1f | 5'-ATGGCTGCTCAACAACAA-3' |
| *MFA1* | MFA1r | 5'-TTACATAACAGAACAAGT-3' |
