# Supplementary material for: Genes Selectively Up-Regulated by Pheromone in White Cells Are Involved in Biofilm Formation in Candida albicans
Source: PLoS Pathog. 2009 Oct 2;5(10):e1000601. doi: 10.1371/journal.ppat.1000601 (PMC2745568; doi:10.1371/journal.ppat.1000601)
Supplement: Table S4 — C. albicans strains used. (0.06 MB DOC) [file ppat.1000601.s006.doc]

|  | **Supporting information** | | | |  | |  | |
| --- | --- | --- | --- | --- | --- | --- | --- | --- |
|  |  |  | | |  | |  | |
|  | **Supplemental Table S4. *C. albicans* strains used.** | | | | | |  | |
|  | | | | | | | |  |
| Strain | | | Parent | *MTL* | | Relevant Genotype | | Reference or source |
| P37005 | | | − | **a**/**a** | | Wild type | | Lockhart *et al.* (2002) |
| WO-1 | | | − | α/α | | Wild type | | Slutsky *et al.* (1987) |
| *eap1/eap1* | | | P37005 | **a**/**a** | | *eap1*Δ::FRT/*eap1*Δ::FRT | | This study |
| *EAP1*_WPREΔ_/*eap1* | | | P37005 | **a**/**a** | | *eap1*Δ::FRT/*EAP1*-WPREΔ::FRT | | This study |
| *EAP1*_WPREΔ_-*EAP1*/*eap1* | | | *EAP1*_WPREΔ_/*eap1* | **a**/**a** | | *eap1*Δ::FRT/ *EAP1*-WPREΔ::FRT-WPRE- *EAP1*::*GFP*::*SAT^R^* | | This study |
| *pga10/pga10* | | | P37005 | **a**/**a** | | *pga10*Δ::FRT/*pga10*Δ::FRT | | This study |
| *PGA10*_WPREΔ_/*pga10* | | | P37005 | **a**/**a** | | *pga10*Δ::FRT/*PGA10*-WPREΔ::FRT | | This study |
| *PGA10*_WPREΔ_-*PGA10*/*pga10* | | | *PGA10*_WPREΔ_/*pga10* | **a**/**a** | | *pga10*Δ::FRT/ *PGA10*-WPREΔ::FRT-WPRE- *PGA10*::*GFP*::*SAT^R^* | | This study |
| *csh1/csh1* | | | P37005 | **a**/**a** | | *csh1*Δ::FRT/*csh1*Δ::FRT | | This study |
| *CSH1*_WPREΔ_/*csh1* | | | P37005 | **a**/**a** | | *csh1*Δ::FRT/*CSH1*-WPREΔ::FRT | | This study |
| *CSH1*_WPREΔ_-*CSH1*/*csh1* | | | *CSH1*_WPREΔ_/*csh1* | **a**/**a** | | *csh1*Δ::FRT/ *CSH1*-WPREΔ::FRT-WPRE- *CSH1*::*GFP*::*SAT^R^* | | This study |
| *pbr1/pbr1* | | | P37005 | **a**/**a** | | *pbr1*Δ::FRT/*pbr1*Δ::FRT | | This study |
| *PBR1*_WPREΔ_/*pbr1* | | | P37005 | **a**/**a** | | *pbr1*Δ::FRT/*PBR1*-WPREΔ::FRT | | This study |
| *PBR1*_WPREΔ_-*PBR1*/*pbr1* | | | *PBR1*_WPREΔ_/*pbr1* | **a**/**a** | | *pbr1*Δ::FRT/ *PBR1*-WPREΔ::FRT-WPRE- *PBR1*::*GFP*::*SAT^R^* | | This study |
| P37005-tet*PBR1* | | | P37005 | **a**/**a** | | *ADH1*/*adh1*Δ::ptet-*PBR1-GFP*::*SAT^R^* | | This study |
| *EAP1*_WPREΔ_/*eap1*-tet*PBR1* | | | *EAP1*_WPREΔ_/*eap1* | **a**/**a** | | *eap1*Δ::FRT/*EAP1*-WPREΔ::FRT *ADH1*/*adh1*Δ::ptet-*PBR1-GFP*::*SAT^R^* | | This study |
| *PGA10*_WPREΔ_/*pga10*-tet*PBR1* | | | *PGA10*_WPREΔ_/*pga10* | **a**/**a** | | *pga10*Δ::FRT/*PGA10*-WPREΔ::FRT *ADH1*/*adh1*Δ::ptet-*PBR1-GFP*::*SAT^R^* | | This study |
| *CSH1*_WPREΔ_/*csh1*-tet*PBR1* | | | *CSH1*_WPREΔ_/*csh1* | **a**/**a** | | *csh1*Δ::FRT/*CSH1*-WPREΔ::FRT *ADH1*/*adh1*Δ::ptet-*PBR1-GFP*::*SAT^R^* | | This study |
| *PBR1*_WPREΔ_/*pbr1*-tet*PBR1* | | | *PBR1*_WPREΔ_/*pbr1* | **a**/**a** | | *pbr1*Δ::FRT/*PBR1*-WPREΔ::FRT *ADH1*/*adh1*Δ::ptet-*PBR1-GFP*::*SAT^R^* | | This study |
| *eap1/eap1*-tet*PBR1* | | | *eap1/eap1* | **a**/**a** | | *eap1*Δ::FRT/*eap1*Δ::FRT *ADH1*/*adh1*Δ::ptet-*PBR1-GFP*::*SAT^R^* | | This study |
| *csh1/csh1*-tet*PBR1* | | | *csh1/csh1* | **a**/**a** | | *csh1*Δ::FRT/*csh1*Δ::FRT *ADH1*/*adh1*Δ::ptet-*PBR1-GFP*::*SAT^R^* | | This study |
| *cek1/cek1 cek2/cek2* | | | P37005 | **a**/**a** | | *cek1*Δ::FRT/*cek1*Δ::FRT *cek2*Δ::FRT/*cek2*Δ::FRT | | Yi *et al.* (2008) |
| *cek1 cek2*-tet*PBR1* | | | *cek1/cek1 cek2/cek2* | **a**/**a** | | *cek1*Δ::FRT/*cek1*Δ::FRT *cek2*Δ::FRT/*cek2*Δ::FRT *ADH1*/*adh1*Δ::ptet-*PBR1-GFP*::*SAT^R^* | | This study |
|  | | |  |  | |  | |  |
