# Supplementary material for: Genes Selectively Up-Regulated by Pheromone in White Cells Are Involved in Biofilm Formation in Candida albicans
Source: PLoS Pathog. 2009 Oct 2;5(10):e1000601. doi: 10.1371/journal.ppat.1000601 (PMC2745568; doi:10.1371/journal.ppat.1000601)
Supplement: Table S5 — The white-specific pheromone response elements (WPRE) found in genes up-regulated by α-pheromone exclusively in white cells and in genes up-regulated by pheromone in both white and opaque cells. (0.06 MB DOC) [file ppat.1000601.s007.doc]

| **Supporting information** | |  |
| --- | --- | --- |
|  |  |  |
| **Table S5. The white-specific pheromone response elements (WPRE) found in genes up-regulated by α-pheromone exclusively in white cells and in genes up-regulated by pheromone in both white and opaque cells.** | | |

**Threshold E value < e-03**

| Gene | WPRE  position | WPRE | P value | WPRE  range | Orientation |
| --- | --- | --- | --- | --- | --- |
| EAP1 | -163 | **AAAAAAAAATAAAGG** | 2.98e-05 | -163 to -149 | - |
| *PGA10* | -140 | **AGAAGAAAATGAAAG** | 2.84e-05 | -140 to -126 | - |
| *CSH1* | -33 | **AAAAAAAAAAACAGG** | 2.49e-05 | -33 to -19 | + |
| *PBR1* | -207 | **AAAACAAAAGGAAAG** | 4.10e-05 | -207 to -193 | + |
| *RBT5* | -316 | **CAAAACAAAACAAAG** | 2.29e-05 | -316 to -302 | - |
| *LSP1* | -197 | **AAAAAAAAAGGAAGG** | 2.02e-05 | -197 to -183 | - |
|  | -491 | **AAAGAAAAGAAAAAG** | 4.79e-05 | -491 to -477 | - |
|  | -444 | **AAGAAAGAAAGAAAG** | 5.25e-05 | -444 to -430 | - |
|  | -174 | **GAAGAAGAAAGAAAG** | 7.59e-05 | -174 to -160 | - |
| *PHR1* | -834 | **AAAAAAAAAACCAAG** | 4.10e-05 | -834 to -820 | + |
|  | -766 | **AAGAAAAAAAAAATG** | 7.98e-05 | -766 to -742 | - |
| *PHR2* | -145 | **AAAAAAAAAAGAAAG** | 2.02e-06 | -145 to -131 | - |
|  | -224 | **AAAAAAAAAACAAAG** | 5.25e-06 | -224 to -210 | - |
|  | -165 | **GAAAGATGAAGAAAG** | 2.85e-04 | -165 to -151 | - |
| *SUN41* | -671 | **AAAAACAAAACAAAG** | 4.10e-05 | -671 to -657 | - |
| *WH11* | -53 | **AAAAAAAAAGGAAGG** | 1.43e-04 | -53 to -39 | - |
| *Orf19.2077* | -94 | **AAAAAAAAAGGAAAG** | 6.99e-05 | -94 to -80 | + |
|  | -432 | **CAAAAAAAACAGAAG** | 2.25e-04 | -432 to -418 | - |
| *CIT1* | -82 | **AAAAAAAAGTGAAAG** | 9.86e-05 | -82 to -68 | - |
|  | -420 | **GGAGAAAAAAAAAAG** | 1.92e-04 | -420 to -406 | - |
|  | -61 | **AGGAAAAGAAGAAAT** | 3.82e-05 | -61 to -47 | - |
| STE2 | -786 | **AAAAAAAAAACCAAC** | 1.85e-04 | -786 to -772 | - |
| *CEK2* | -200 | **AAAAAAATAAAAAAA** | 3.43e-04 | -200 to -186 | + |
| *SST2* | -323 | **AAAGAAACCAAAAAA** | 4.61e-04 | -323 to -309 | - |
| *RBT1* | -706 | **AGAAAAAACAGAAAG** | 2.66e-05 | -706 to -692 | + |
| Consensus | | **AAAAAAAAAAGAAAG** |  |  |  |

Threshold e-03 < E value < e-02

| Gene | WPRE  position | WPRE | P value | WPRE  range | Orientation |
| --- | --- | --- | --- | --- | --- |
| EAP1 | -500 | **AAAAAAAACATAAAC** | 4.03e-03 | -500 to -486 | + |
| *PGA10* | -432 | **GGAAACAAGACCAAG** | 6.17e-03 | -432 to -418 | - |
| *CSH1* | -408 | **GAAACAGACGGAAGG** | 3.32e-03 | -408 to -394 | + |
| *PBR1* | -262 | **CTAAAAAAAAAAAAG** | 1.35e-03 | -262 to -248 | - |
| Consensus | | **AAAAAAAAAAGAAAG** |  |  |  |
