# Supplementary material for: Genes Selectively Up-Regulated by Pheromone in White Cells Are Involved in Biofilm Formation in Candida albicans
Source: PLoS Pathog. 2009 Oct 2;5(10):e1000601. doi: 10.1371/journal.ppat.1000601 (PMC2745568; doi:10.1371/journal.ppat.1000601)
Supplement: Table S6 — The opaque-specific pheromone response elements (OPRE) found in genes up-regulated by α-pheromone exclusively in opaque cells and in genes up-regulated by pheromone both in white and opaque cells. (0.06 MB DOC) [file ppat.1000601.s008.doc]

**Supporting Information**

**Table S6. The opaque-specific response elements (OPRE) found in genes up-regulated by α-pheromone exclusively in opaque cells and in genes up-regulated by pheromone both in white and opaque cells.**

**Threshold E value < e-03**

| Gene | OPRE  Position | OPRE | P value | OPRE  range | Orientation |
| --- | --- | --- | --- | --- | --- |
| MFA1 | -266 | **GTGAGGGGG** | 1.23e-06 | -266 to -258 | + |
| *FUS1* | -110 | **GTGGGGGGG** | 1.59e-04 | -110 to -102 | + |
| *CPH1* | -364 | **GTGGGGGGA** | 8.69e-06 | -364 to -356 | - |
| *ECE1* | -946 | **GCGAGGCGA** | 5.70e-04 | -946 to -938 | - |
| *KAR4* | -302 | **GCGTGCGTG** | 1.70e-04 | -302 to -294 | + |
| *RAM1* | -319 | **GAGAGGGGA** | 1.32e-06 | -319 to -311 | - |
| STE2 | -176 | **GGGAGGGGG** | 4.87e-05 | -176 to -168 | - |
| *CEK2* | -183 | **GGAAGGGGA** | 2.63e-06 | -183 to -175 | - |
| *SST2* | -246 | **GGGGGAGGG** | 1.44e-05 | -246 to -238 | - |
| *RBT1* | -918 | **CTGGAGGGA** | 3.91e-06 | -918 to -910 | - |
| Consensus | | **GTGAGGGGA** |  |  |  |

Threshold e-03 < E value < e-02

| Gene | OPRE  Position | OPRE | P value | OPRE  range | Orientation |
| --- | --- | --- | --- | --- | --- |
| *CPH1* | -54 | **ACGAGGGGG** | 5.37e-03 | -54 to -46 | - |
| MFA1 | -392 | **GTGAGCGTA** | 1.24e-03 | -392 to -384 | + |
| *SST2* | -267 | **GGGGGAGGG** | 3.78e-03 | -267 to -259 | - |
| Consensus | | **GTGAGGGGA** |  |  |  |

**Threshold E value = 4 ×** e+02

| Gene | OPRE  Position | OPRE | P value | OPRE  range | Orientation |
| --- | --- | --- | --- | --- | --- |
| MFA1 | -963 | **GAAAGAAAAAACAAA** | 6.54e+02 | -963 to -949 | + |
| *FUS1* | -267 | **GCAACCAAAAAAAAA** | 1.96e+01 | -267 to -253 | + |
| *CPH1* | -806 | **GAAACAAAAACAAAA** | 5.83e+02 | -806 to -792 | - |
| *ECE1* | -449 | **GACGCCAAGAAAAAA** | 3.66e+03 | -449 to -435 | + |
| *KAR4* | -95 | **AAAACCAAAAAAGAG** | 2.41e+02 | -95 to -81 | - |
| *RAM1* | -566 | **GAAACCAAAATACAA** | 1.33e+01 | -566 to -552 | - |
| STE2 | -787 | **GAAAAAAAAAAAAAA** | 4.76e+02 | -787 to -773 | + |
| *CEK2* | -47 | **GAAAATAAAAAAAAA** | 2.09e+02 | -47 to -33 | - |
| *SST2* | -329 | **GAAACCAAAAAAAAA** | 1.02e+01 | -329 to -315 | + |
| *RBT1* | -896 | **GCAGGCAAAAAAAAA** | 1.45e+03 | -896 to -882 | - |
| Consensus | | **GAAACCAAAAAAAAA** |  |  |  |
