# Supplementary material for: Genes Selectively Up-Regulated by Pheromone in White Cells Are Involved in Biofilm Formation in Candida albicans
Source: PLoS Pathog. 2009 Oct 2;5(10):e1000601. doi: 10.1371/journal.ppat.1000601 (PMC2745568; doi:10.1371/journal.ppat.1000601)
Supplement: Table S7 — The significance of the differences in biofilm thickness between complemented controls and deletion mutants, described in Figure 6. (0.04 MB DOC) [file ppat.1000601.s009.doc]

|  | Versus complemented control, p value | |
| --- | --- | --- |
| Mutant | -Op | +Op |
|  |  |  |
| *eap1/eap1* | 4x10^-6^ | 2x10^-10^ |
| *EAP1_WPREΔ_/eap1* | 1x10^-6^ | 2x10^-10^ |
|  |  |  |
| *pga10/ pga10* | 4x10^-10^ | 5x10^-9^ |
| *PGA10_WPREΔ_/pga10* | 2x10^-5^ | 4x10^-10^ |
|  |  |  |
| *csh1/csh1* | 1x10^-2^ | 6x10^-11^ |
| *CSH1_WPREΔ_/csh1* | 8x10^-9^ | 5x10^-11^ |
|  |  |  |
| *pbr1/pbr1* | 2x10^-2^ | 6x10^-8^ |
| *PBR1_WPREΔ_/pbr1* | 7x10^-3^ | 9x10^-13^ |

Table S7. The significance of the difference in biofilm thickness between complemented controls and deletion mutants, described in Figure 6.
